# Effects of exercise and physical activity on circulating inflammaging- and immunosenescence-related biomarkers in older adults and adults with chronic diseases: an umbrella review

**DOI:** 10.3389/fimmu.2026.1931607

**Published:** 2026-09-29

**Authors:** Guangwei Chai, Jinhong Xue, Bin Xie

**Affiliations:** 1 Department of Sports Human Science, Hebei Sport University, Shijiazhuang, China; 2 Department of Wushu, Hebei Sport University, Shijiazhuang, China; 3 Department of Physical Education, Hebei Chemical & Pharmaceutical College, Shijiazhuang, Hebei, China

**Keywords:** ageing, chronic disease, cytokines, exercise, immunosenescence, inflammaging, physical activity, umbrella review

## Abstract

**Background:**

Chronic low-grade inflammation and immunosenescence accompany ageing and chronic disease. Exercise and physical activity are proposed to modify circulating inflammatory biomarkers, but review-level evidence is dispersed across populations and conditions.

**Methods:**

We searched PubMed, Web of Science, Embase, Cochrane Library, Scopus, and SPORTDiscus through 1 March 2026 for systematic reviews, meta-analyses, and overviews on exercise or physical activity and circulating inflammaging- or immunosenescence-related biomarkers in older adults and adults with chronic diseases. We reported the review in accordance with PRISMA 2020 and PRIOR. Methodological quality was appraised with A MeaSurement Tool to Assess systematic Reviews, version 2 (AMSTAR-2), primary-study overlap with the corrected covered area, and certainty with GRADE. We performed no *de novo* meta-analysis or meta-regression; this umbrella review is a structured narrative synthesis of quantitative review-level estimates rather than a quantitative reanalysis.

**Results:**

Twenty-one reviews were included: 14 core-eligible (full Population, Intervention, Comparator, Outcomes, and Study design [PICOS] match, contributing to the primary synthesis and conclusions) and seven evidence-map-only (partial PICOS match, used only for mapping and background). Among core-eligible reviews reporting standardised mean differences, exercise was associated with small reductions in tumour necrosis factor-alpha (TNF-alpha; point estimates − 1.42 to − 0.05, two of five confidence intervals excluding the null) and C-reactive protein (CRP; − 0.65 to − 0.47, two of three excluding the null), whereas interleukin-6 (IL-6) estimates were inconsistent (− 0.81 to + 0.09, two of five excluding the null). Heterogeneity was frequently high (*I*^2^ up to 94%). Overall primary-study overlap was slight (corrected covered area [CCA] = 0.0091), but very high within the multiple sclerosis cluster (CCA = 0.4048). AMSTAR-2 confidence was critically low in 11 reviews, low in nine, and moderate in one; GRADE certainty was very low for every biomarker construct. A *post hoc* restricted descriptive sensitivity analysis excluding critically low reviews attenuated the apparent signal.

**Conclusion:**

Exercise and physical activity may produce small reductions in some circulating inflammatory biomarkers, most consistently TNF-alpha and CRP, but effects on IL-6 are inconsistent, and certainty is very low. Effects on immunosenescence and cellular-senescence markers remain an evidence gap. This biomarker evidence alone is insufficient to justify prescribing exercise specifically to lower inflammatory biomarkers and does not alter broader physical-activity recommendations based on clinical and functional outcomes.**PROSPERO**: CRD420261410061

## Introduction

Ageing is accompanied by a chronic, low-grade, sterile inflammatory state termed “inflammaging”, characterised by modest but persistent elevations in circulating proinflammatory mediators (1). This phenotype was originally framed as an evolutionary trade-off in which lifelong antigenic and stress exposure sustains a proinflammatory network that becomes maladaptive in later life (1). Inflammaging is now recognised as a shared mechanism linking ageing to cardiovascular disease, frailty, sarcopenia, cognitive decline, metabolic disease, and cancer, and elevated interleukin-6 (IL-6), tumour necrosis factor-alpha (TNF-α), and C-reactive protein (CRP) predict disability and mortality in older people (2, 3). Chronic inflammation and immune dysregulation feature prominently among the hallmarks of ageing (4). Inflammaging is closely intertwined with immunosenescence—the age-related remodelling of innate and adaptive immunity—such that the two processes reinforce one another (5).

A parallel driver of inflammaging is cellular senescence: senescent cells accumulate with age and secrete a proinflammatory “senescence-associated secretory phenotype” that includes IL-6, IL-8, CRP, and chemokines such as monocyte chemoattractant protein-1 (CCL2/MCP-1), and can be marked by cell-cycle regulators such as cyclin-dependent kinase inhibitor 2A (p16INK4a) and p21Cip1 (4). Circulating cytokines and acute-phase proteins are therefore widely used as accessible, if nonspecific, biomarkers of the inflammaging–senescence axis, although their interpretation is complicated by biological pleiotropy (3).

Regular physical activity is one of the few interventions plausibly able to modify this axis. Contracting skeletal muscle behaves as an endocrine organ, releasing myokines that exert systemic anti-inflammatory effects, and habitual exercise is associated with lower resting concentrations of several inflammatory mediators and with better-preserved immune function in older adults (6–9). IL-6 occupies a paradoxical position in this framework: it is markedly and transiently released from working muscle as a myokine that triggers downstream anti-inflammatory signalling, yet chronically elevated resting IL-6 is a hallmark of the low-grade inflammation of ageing and disease — so changes in resting IL-6 after training are inherently difficult to interpret (6, 8). International guidelines recommend regular aerobic and muscle-strengthening activity for older adults and people with chronic conditions on the basis of clinical and functional outcomes such as mortality, cardiometabolic health, physical function and mental health rather than inflammatory biomarkers (10). The anti-inflammatory and immunoprotective effects described above are proposed mechanisms supported by exercise-immunology literature, not a stated basis of those guideline recommendations (6, 7, 9).

Many systematic reviews and meta-analyses have examined exercise and circulating inflammatory biomarkers, but they are fragmented across populations and modalities, use different effect metrics and comparators, and reach heterogeneous conclusions; overlapping reviews of the same primary trials can also create a false impression of independent replication (11). Mechanistic and narrative reviews likewise describe anti-inflammatory pathways (6, 7, 9), but such statements are not equivalent to clinical guidance to prescribe exercise specifically for biomarker reduction. Whether the review-level evidence supports that inference after considering methodological quality, primary-study overlap, and certainty has not been examined. An umbrella review can map and appraise this dispersed evidence, quantify overlap, and summarise what can and cannot be concluded. We therefore conducted an umbrella review to determine what systematic-review evidence shows about exercise or physical activity and circulating inflammatory/inflammaging biomarkers and extractable ageing-related markers in older adults and adults with chronic diseases. The main purpose was to establish what the existing review-level evidence can and cannot support; priorities for future trials were derived from the gaps identified by this appraisal.

## Methods

### Protocol, registration, and reporting

This umbrella review was conducted and reported in accordance with the Preferred Reporting Items for Systematic Reviews and Meta-Analyses (PRISMA) 2020 statement (12) and the Preferred Reporting Items for Overviews of Reviews (PRIOR) statement (13), and the literature search was reported following PRISMA-S (14). The review was registered in PROSPERO during conduct (CRD420261410061; first posted 30 May 2026), after the formal search had been completed and while screening was underway. The initial record was created within the same research team, and the authors were listed as Yiqiang Wang and Weibao Liang, and used a working title focused on T-cell immune remodelling within a broader exercise–immune-ageing framework. Responsibilities were subsequently reorganised, and Guangwei Chai and Jinhong Xue assumed responsibility for the present review and manuscript, whose reporting focus was refined to circulating inflammaging- and immunosenescence-related biomarkers. The registered secondary outcomes included inflammatory cytokines such as IL-6 and TNF-α; the target populations, exercise/physical-activity exposure, review-level design, methodological appraisal, primary-study-overlap assessment, and certainty framework remained applicable. The executed search used SPORTDiscus rather than PsycINFO and applied an English-language limit where supported by database interfaces. These differences, together with the final title and review team, are reported transparently as amendments to the protocol-stage record. The PROSPERO record has been updated so that it accurately reflects the review as conducted and reported; the original version history is preserved and remains part of the audit trail.

### Eligibility criteria

We included systematic reviews, meta-analyses, network meta-analyses, Bayesian meta-analyses, dose-response meta-analyses, and overviews/umbrella reviews of human intervention or exposure evidence. Eligible populations comprised two complementary categories: older adults (aged ≥ 60/≥ 65 years or as defined by review authors) and adults with chronic diseases irrespective of an older-age threshold. These categories were not treated as statistically interchangeable age strata. Chronic disease can be accompanied by inflammatory and immune-ageing phenotypes before age 60 (2, 3, 5), and excluding disease-defined adults would have answered a substantially narrower question. Age and diagnosis were therefore retained as contextual descriptors, and the resulting heterogeneity was treated as a limitation on generalisability. Reviews containing participants younger than 18 years were core-eligible only when adult-only estimates were separable; otherwise, they were retained for the evidence map only. Eligible interventions/exposures were aerobic exercise, resistance training, combined exercise, structured physical activity, walking/exercise rehabilitation, yoga, and acute or chronic exercise training. Whole-body vibration was core-eligible only when it was one modality within a broader active-exercise scope; reviews restricted to stand-alone vibration exposure were evidence-map-only because that exposure was not directly comparable with active aerobic or resistance exercise. Comparators were usual care, no-exercise control, non-whole-body-vibration control, preintervention baseline, lower physical activity/reference groups, or the comparator reported by each source review. Core outcomes were circulating IL-6, TNF-α, CRP/high-sensitivity CRP (hs-CRP), interleukin-1beta (IL-1β), interleukin-8/CXCL8 (IL-8), CCL2/MCP-1, p16INK4a, p21Cip1, and telomere/telomerase when truly extracted; related markers such as interleukin-18 (IL-18) were retained when directly reported.

Inclusion in the main analysis required that a review had genuinely extracted or pooled at least one core biomarker, rather than mentioning it only as background. Reviews limited to cellular immune counts/function without core-biomarker extraction, reviews in which IL-6/TNF-α appeared only as narrative background, animal-only evidence, purely meditation/mindfulness interventions without separable physical activity, and mRNA-only evidence were outside the main-effect extraction. Original studies were not units of analysis and were used only to trace primary-study overlap. Each review was classified as core-eligible (full PICOS match; contributing to the primary analysis and main conclusions) or evidence-map-only (partial PICOS match; used only for the evidence map and background); evidence-map-only results are labelled throughout and do not support the main conclusions.

### Information sources and search

We searched PubMed, Web of Science Core Collection, Embase, Cochrane Library, Scopus, and SPORTDiscus from inception through 1 March 2026, limited to human studies and English-language records where the interface allowed. Search strings combined controlled vocabulary (MeSH/Emtree/subject headings) and free-text terms for four concept blocks—population, intervention/exposure, outcome, and study design (systematic reviews and meta-analyses)—with the Boolean operator AND. The full, database-specific strategies as executed are provided in **Supplementary Table 1**.

### Study selection and data extraction

Two reviewers independently screened titles/abstracts and full-text reports against the eligibility criteria and independently extracted data from each included review using a piloted form. Disagreements were resolved by consensus, with third-reviewer consultation if consensus could not be reached. Extracted items comprised review characteristics; outcome-level effect estimates (effect size, 95% confidence or credible interval, effect metric, *I*^2^, number of contributing primary studies, participants, comparator, and timepoint); the risk-of-bias tool used for the primary studies; and the certainty framework reported. All extracted quantitative values were verified against the text, tables, and figures of the source reviews, and discrepancies were resolved by returning to the source. Numerical values are reported as extracted and reproduced from the source reviews. Full outcome-level records, including primary, subgroup, sensitivity, narrative-only, and evidence-map-only results, are provided in **Supplementary Table 2**.

### Methodological quality of included reviews

The methodological quality of each included review was appraised using A MeaSurement Tool to Assess systematic Reviews, version 2 (AMSTAR-2), which rates overall confidence as high, moderate, low, or critically low on the basis of weaknesses in seven critical domains (protocol registration, adequacy of the search, justification of excluded studies, risk-of-bias assessment, appropriateness of meta-analytic methods, consideration of risk of bias when interpreting results, and assessment of publication bias) (15). Item-level ratings for all reviews are displayed as a traffic-light figure, and overall confidence together with the principal AMSTAR-2 limitations are summarised in the main tables. A critical domain was counted as a critical flaw only when the corresponding item was rated “no”; a rating of “partial yes” in a critical domain was recorded as a non-critical weakness and did not by itself lower overall confidence.

### Primary-study overlap

Overlap of primary studies across reviews was quantified with the corrected covered area (CCA), computed from a study-by-review citation matrix (16); CCA values were interpreted using established bands (slight 0%–5%, moderate > 5%–10%, high > 10%–15%, very high > 15%) (17). Overlap was examined overall and within predefined disease/intervention clusters. High overlap invalidates cross-review pooling, as the same primary study would otherwise be counted more than once; therefore, reviews within high-overlap clusters were summarised rather than combined.

### Certainty of evidence

Certainty was assessed with the GRADE approach and summarised descriptively at the construct (domain) level across contributing reviews (18). We distinguished certainty reported by a source review (SR-reported) from certainty applied for this umbrella review from the available information (reviewer-applied); reviewer-applied grading drew on primary-study risk of bias, inconsistency, indirectness, imprecision and publication bias where these could be judged. Indirectness reflected partial alignment with the review question, including non-separable ineligible populations or evidence-map-only scope; differences in adult age alone were not automatically downgraded and are addressed primarily as a limit on generalisability (**Supplementary Table 3**). Each review contributed at most one reviewer-applied row per construct. When a review supplied multiple within-review subestimates, we retained the most conservative certainty grade and the most severe judgement in each GRADE domain; a separate source-reported rating, where available, was retained as an additional entry (**Supplementary Table 4**). Where reviews gave different grades for the same construct, we report the range and the most conservative grade. To avoid a proliferation of single-study fragments, certainty is presented per construct rather than per individual estimate.

### Data synthesis

We did not perform a *de novo* meta-analysis at the umbrella level. This review is quantitative in its inputs and narrative in its synthesis: review-level estimates were reproduced, harmonised in direction, tabulated, and appraised, but no umbrella-level pooling, meta-regression or dose-response modelling was performed, and no new summary effect size is reported. Numerical ranges therefore describe what the existing reviews reported and are not pooled effect estimates. Repooling had been prespecified only if overlap was low, effect metrics and populations were sufficiently homogeneous, and the analysis was preplanned rather than exploratory. These conditions were not met: metrics differed (standardised mean difference [SMD] versus mean difference [MD]), populations were clinically heterogeneous, and overlap was very high within the multiple sclerosis cluster. In addition, the predominance of low or critically low AMSTAR-2 confidence further argued against any *post hoc* repooling. Effect directions were harmonised so that a negative estimate favours exercise for all biomarkers. SMD and MD estimates were never combined, and publication bias or small-study effects were reported only as assessed by source reviews. For the evidence map, one result was selected per review–construct pair: overall analyses were preferred to subgroup or sensitivity analyses, and controlled between- group comparisons were preferred to within-person or cross-sectional comparisons when multiple eligible results remained. The evidence-map columns indicate direction only and do not compare effect magnitude across metrics. We additionally performed a *post hoc* restricted descriptive sensitivity analysis excluding core-eligible reviews rated critically low on AMSTAR-2. No pooled effect was computed; we compared the remaining ranges and the number of intervals excluding the null. AMSTAR-2 was used only as a review-level restriction and not as a substitute for primary-study risk-of-bias assessment (**Supplementary Table 5**).

## Results

### Review selection

The selection process is summarised in the PRISMA 2020 flow diagram (**Figure 1**). Database searches identified 1,498 records; after 853 duplicates were removed, 645 records were screened, of which 457 were excluded. Of 188 full-text reports assessed for eligibility, 167 were excluded, leaving 21 systematic reviews (19–39). Following an outcome-eligibility audit, Baker et al. (19) (R01) was reclassified as evidence-map-only because its source meta-analysis included a paediatric Crohn disease trial and did not provide separable adult-only pooled estimates. The total number of included reviews was unchanged; 14 reviews were core-eligible, and seven were evidence-map-only.

**Figure 1:**
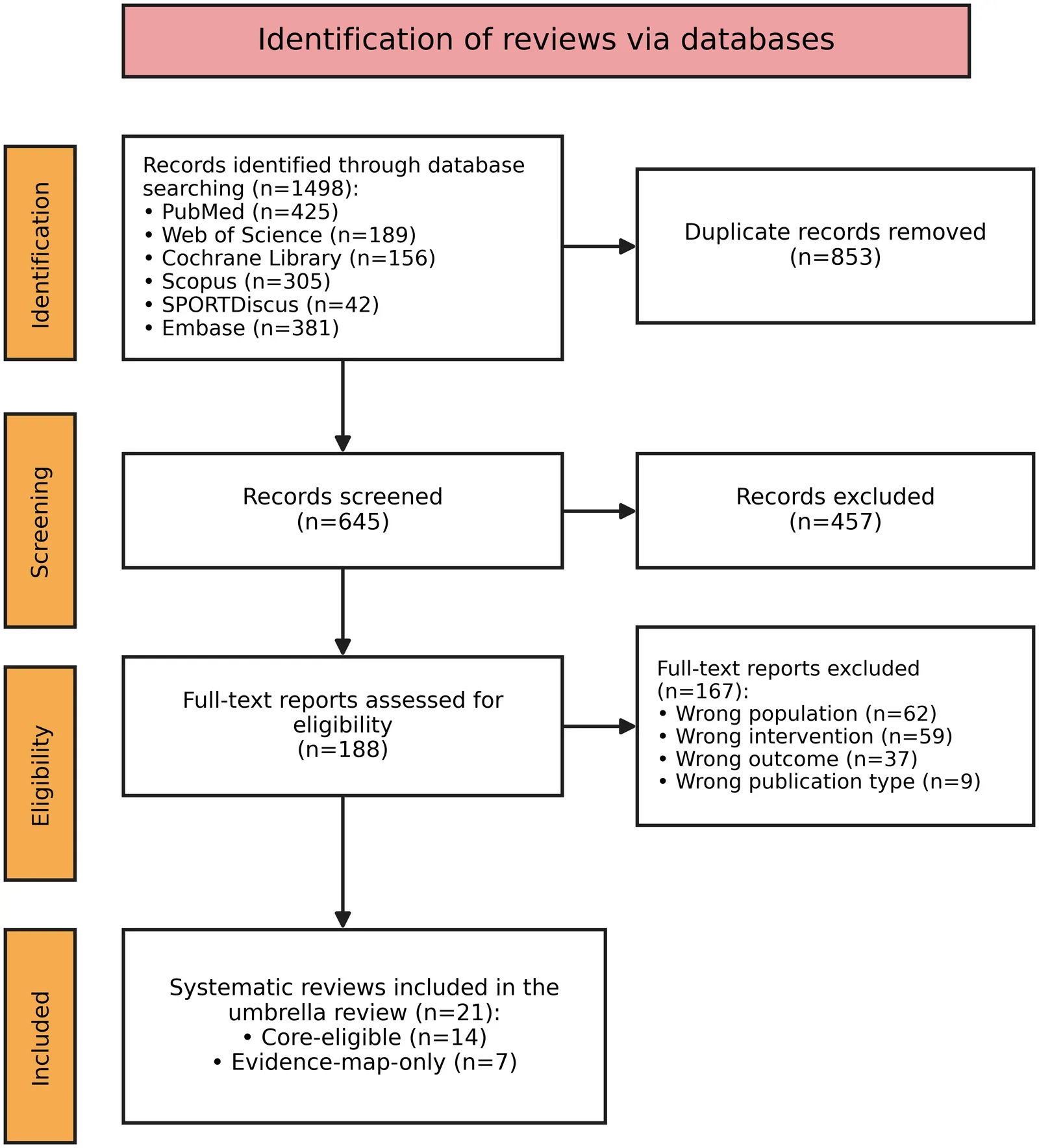
PRISMA 2020 flow diagram of study selection. The diagram shows the identification, screening, eligibility, and inclusion of systematic reviews from six databases.

### Characteristics of included reviews and primary-study overlap

The 21 reviews were published between 2008 and 2026 and spanned multiple sclerosis (R05, R10, R11, R20), cancer (R06) or breast cancer (R18), HIV (R13, R21), heart failure with overweight/obesity (R07), dementia or mild cognitive impairment (R16), frailty/sarcopenia (R02), inflammatory bowel disease (R01), vaccination and physical activity (R12), whole-body vibration (R19), senolytic/ageing biology (R03), and general or healthy older adults (R04, R09, R15, R17), with one nested overview (R14). Review types ranged from narrative systematic reviews to meta-analyses, including a Bayesian meta-analysis (R15). Interventions encompassed aerobic, resistance, combined, and concurrent training, walking or exercise rehabilitation, yoga, whole-body vibration, and structured physical activity, generally compared with usual care, no-exercise control, or preintervention baseline. Primary-study risk of bias was most often assessed with PEDro-based tools, Cochrane risk-of-bias tools, or the Jadad scale, and was not reported in three reviews. Age criteria differed substantially: five reviews required participants aged at least 60 years or a mean age of at least 60 years, one used a threshold of 50 years, and disease-specific reviews generally included younger and middle-aged adults (**Supplementary Table 3**). Full characteristics are given in **Table 1**.

**Table 1:** Characteristics of the included systematic reviews.

Review	Condition/population	Review type	Intervention scope	Comparator	Primary studies	CCA-eligible (*n*)	Participants	Primary-study RoB tool	Evidence tier
Baker et al. (19) (R01)	IBD	SR + MA	Acute and chronic aerobic/resistance or combined exercise interventions	Control/no exercise or pre–postcomparisons as reported	5 in meta-analysis	5	NR	NR	Map
Byrne et al. (20) (R02)	Frailty/sarcopenia	SR + MA	Exercise-based and mixed interventions including nutritional components	Control/usual care or comparator as reported	16	16	NR	Bespoke study-quality framework reported by review	Map
Chen et al. (21) (R03)	Senolytic/aging human	SR	Habitual physical activity and exercise training	Lower physical activity/reference groups or baseline/control	21	8	535 human participants	AUB KQ1 and other study-design-specific quality tools reported	Map
Haaland et al. (22) (R04)	Healthy older adults	SR	Regular aerobic and resistance exercise interventions	Control/no exercise or comparator as reported	19 articles representing 17 trials	17	NR	Jadad scale	Core
Hejazi et al. (23) (R05)	Multiple sclerosis	SR + MA of RCTs	Exercise training interventions	Control/usual-care groups	19 (19 RCTs)	19	569	PEDro	Core
Khosravi et al. (24) (R06)	Cancer	MA	Exercise training interventions	Control/no-exercise groups	27 trials reported in main text	26	NR	Modified PEDro scale	Core
Malandish and Gulati et al. (25) (R07)	Heart failure/overweight-obesity	SR + MA of RCTs	Aerobic, resistance, and combined exercise interventions	Usual care/no-exercise/control groups	46 full-text articles (46 RCTs)	46	3,693	PEDro-based quality assessment	Core
Mishra et al. (26) (R08)	Yoga/mixed conditions	SR + MA	Yoga interventions	Control or comparator groups as reported	26 (26 RCTs)	26	2,091	RoB 2	Map
Monteiro-Junior et al. (27) (R09)	General older adults	SR + MA	Chronic exercise interventions	Control/no-exercise groups	8	8	NR	NR	Core
Najafi et al. (28) (R10)	Multiple sclerosis	SR of randomised clinical trials	Exercise interventions	Control/usual-care groups or pre–postcomparisons as reported	22 (22 RCTs)	22	742	JBI Critical Appraisal Checklist for RCTs	Core
Negaresh et al. (29) (R11)	Multiple sclerosis	SR	Endurance, resistance, combined and vibration exercise training	Control/pre–postcomparisons as reported	12	12	NR	PEDro scale	Core
Palotino-Ferreira et al. (30) (R12)	Vaccination/physical activity	SR	Structured physical activity	Control/usual activity or comparator as reported	7	7	1,149	TESTEX	Map
Pedro et al. (31) (R13)	HIV	SR	Aerobic, resistance, and concurrent training	Control group or other physical training as reported	5 (5 RCTs)	5	NR	Jadad scale	Core
Reis et al. (32) (R14)	Nested overview	Overview of systematic reviews	Aerobic, resistance, and combined exercise	Comparator as reported by underlying reviews	13 systematic reviews analysed	NA	249–1,421 participants across reviews	Tool names reported by included systematic reviews	Map
Sánchez-Romero et al. (33) (R15)	SASP older adults	SR and Bayesian MA of RCTs	Structured exercise interventions, mainly resistance training with or without aerobic exercise	Usual care/maintenance of usual lifestyle	3 (3 RCTs)	3	1,447	Cochrane RoB 2.0	Core
Stigger et al. (34) (R16)	Dementia/MCI	SR + MA	Physical exercise, especially aerobic exercise	No aerobic/resistance exercise; balance, tone or stretching controls	8 (8 RCTs)	8	NR	Cochrane risk of bias tool	Core
Teodoro et al. (35) (R17)	Older adults TH1/TH2	SR	Acute and chronic exercise	Baseline/control or comparator as reported	14 (RCTs and prospective observational studies)	14	525	Cochrane RoB 2	Core
Ubaghs et al. (36) (R18)	Breast cancer	SR + MA	Aerobic, resistance, combined, yoga/tai chi, HIIT, and other exercise interventions	Usual care/control groups	20 (20 RCTs)	19	1.109	RoB 2	Core
Wang et al. (37) (R19)	Whole-body vibration	MA	Whole-body vibration exercise	Non-WBV controls and post–precomparisons	31 total studies	16	NR	PEDro scale	Map
Wong and Holahan et al. (38) (R20)	Multiple sclerosis	SR	Aerobic, resistance, or combined exercise interventions	Control/baseline or comparator as reported	9	9	NR	Standardized framework	Core
Zanetti et al. (39) (R21)	HIV	SR + MA of RCTs	Resistance training	Control/no resistance training	7 (7 RCTs)	6	258 completed follow-up	RoB 2	Core

Evidence tier: Core = core-eligible (full PICOS match; contributes to the primary synthesis and main conclusions); Map = evidence-map-only (partial PICOS match; used for evidence mapping/background only). Reviews are ordered by review identifier. Baker et al. (19) (R01) was reclassified as Map because its pooled estimates included a paediatric Crohn disease trial and adult-only pooled estimates were not separable. The “Primary studies” column reproduces the count reported by each source review. The “CCA-eligible (*n*)” column gives the number of primary studies that could be individually traced and de-duplicated from the source reference list and were therefore used in the corrected-covered-area analysis (**Figure 2**; **Supplementary Table 6**). The two columns differ when a source count included preclinical or non-human records (R03, R19), when two or more reports of the same trial were de-duplicated to a single primary study (R21), or when a cited record could not be resolved to a distinct, individually citable human primary study (R06, R18). For the nested overview (R14), the units of analysis are systematic reviews rather than primary studies, so no CCA-eligible count applies (NA). Age criteria and reported participant ages are provided in **Supplementary Table 3**.

Primary-study overlap across reviews was slight overall (CCA = 0.0091; 20 reviews, 249 deduplicated primary studies; the nested overview R14 was excluded from the primary-study overlap calculation) but differed markedly by cluster (**Figure 2**). Overlap was very high within the multiple sclerosis cluster (CCA = 0.4048; four reviews, 28 deduplicated studies), with the largest pairwise overlaps between Hejazi et al. (23) and Najafi et al. (28) (13 shared studies) and between Najafi et al. (28) and Negaresh et al. (29) (12 shared studies); it was moderate in the cancer cluster (CCA = 0.0714) and slight or zero in the HIV, general-ageing, and sensitivity clusters (**Supplementary Table 6**). This very high overlap within the multiple sclerosis cluster is a principal reason review-level estimates were summarised rather than pooled.

**Figure 2:**
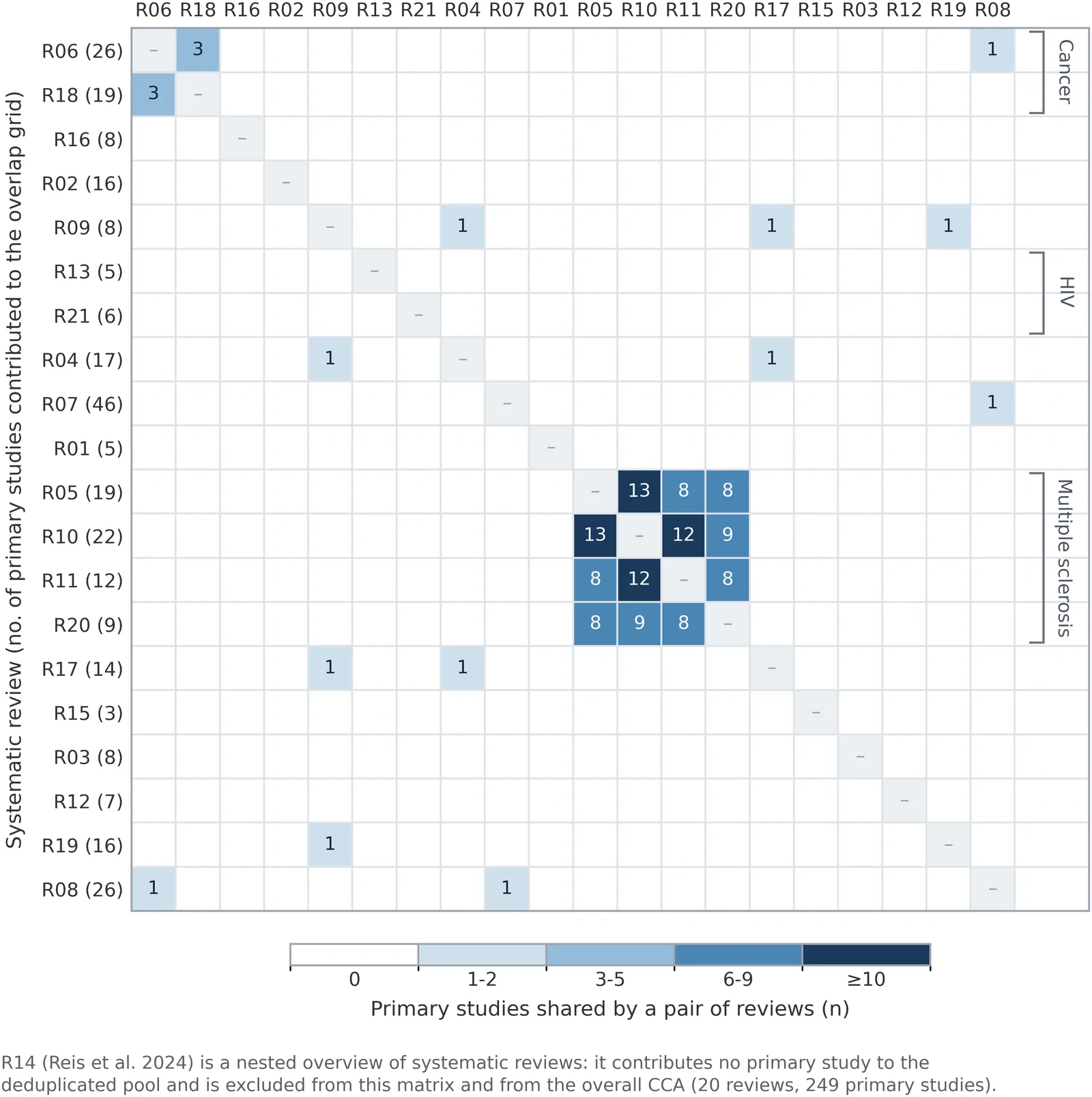
Pairwise primary-study overlap between the included reviews. Pairwise overlap of primary studies across the included systematic reviews, derived from the corrected-covered-area study-by-review grid. Each cell shows the number of primary studies shared by a pair of reviews; darker shading denotes greater overlap. Row labels show the number of CCA-eligible primary studies contributed by each review (**Table 1**); the diagonal is not counted as overlap. Overall CCA was 0.0091 (slight), but the multiple sclerosis cluster showed very high overlap (CCA 0.4048). CCA, corrected covered area.

### Effects of exercise on core inflammatory biomarkers

Across core-eligible reviews, effects of exercise on the three most frequently reported biomarkers (IL-6, TNF-α, and CRP) were small and, for IL-6, inconsistent (**Figure 3**; **Table 2**).

**Figure 3:**
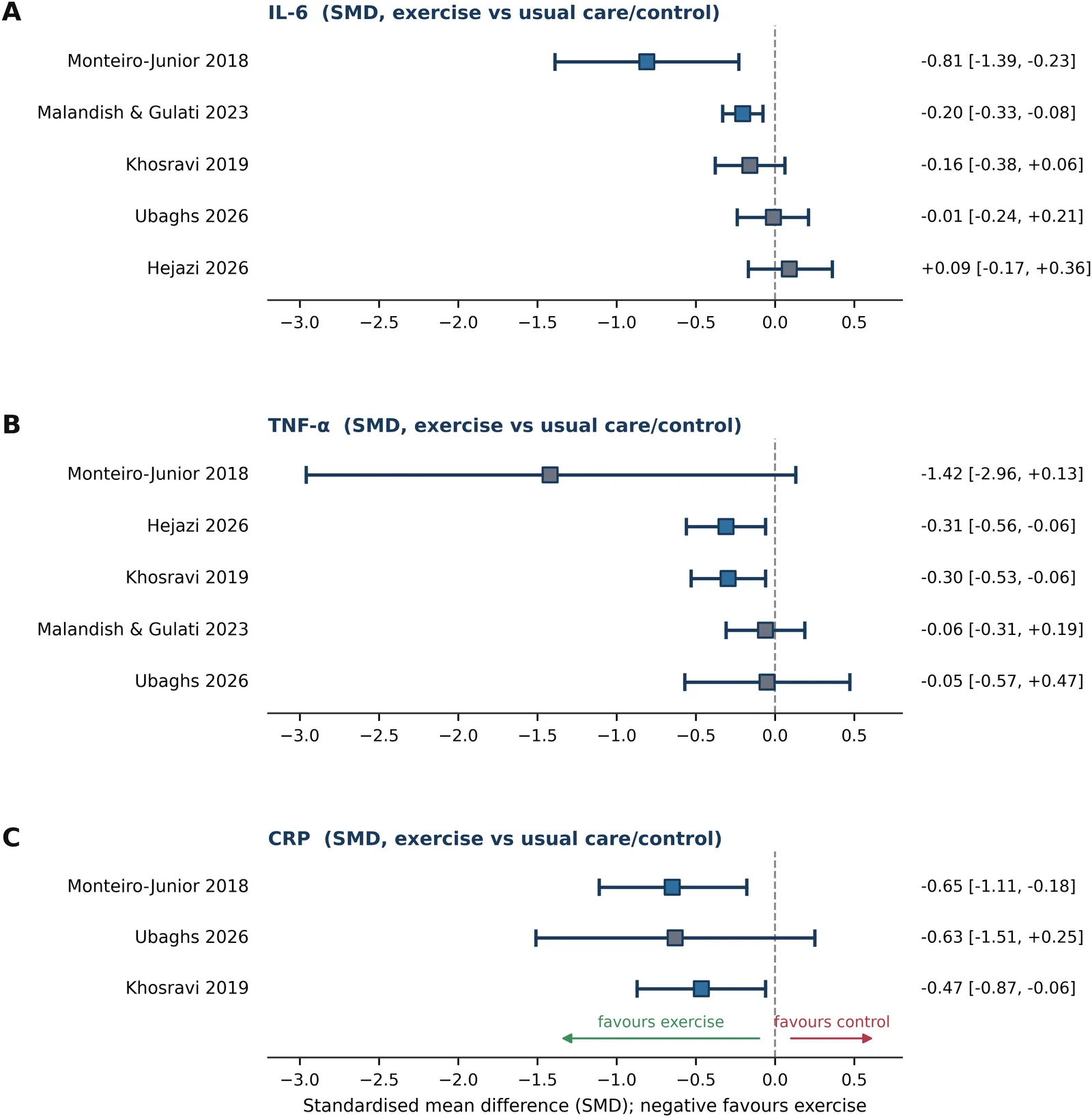
Forest plot of systematic-review-level effect estimates for core inflammatory biomarkers. Forest plot of systematic-review-level standardised mean differences (SMD; exercise/physical activity vs. usual care, no exercise, or control) for **(A)** IL-6, **(B)** TNF-alpha, and **(C)** C-reactive protein. Each row is one systematic review; squares are point estimates (filled where the 95% confidence interval excludes the null, grey where it crosses the null), and whiskers are 95% confidence intervals. Estimates are shown as reported by each review and are not pooled across reviews (see main text). Only core-eligible reviews are plotted; reviews reporting the mean-difference metric are summarised in **Table 2**. A negative value favours exercise. CI, confidence interval; CRP, C-reactive protein; IL-6, interleukin-6; SMD, standardised mean difference; TNF-alpha, tumour necrosis factor alpha.

**Table 2:** Summary of meta-analytic findings for core inflammatory biomarkers (core-eligible reviews).

Review	Condition/population	Outcome (construct)	Metric	*k* (studies/arms)	Effect [95% interval]	*I* ^2^
Sánchez-Romero et al. (33) (R15)	SASP older adults	IL-6	MD	2	− 0.04 [− 0.97, + 0.91]	NR
Stigger et al. (34) (R16)	Dementia/MCI	IL-6	MD	2	− 0.71 [− 0.88, − 0.54]	0%
Zanetti et al. (39) (R21)	HIV	IL-6	MD	2	− 1.18 [− 3.71, + 1.35]	93%
Ubaghs et al. (36) (R18)	Breast cancer	IL-6	SMD	6	− 0.01 [− 0.24, + 0.21]	27%
Khosravi et al. (24) (R06)	Cancer	IL-6	SMD	NR	− 0.16 [− 0.38, + 0.06]	38%
Malandish and Gulati et al. (25) (R07)	Heart failure/overweight-obesity	IL-6	SMD	NR	− 0.20 [− 0.33, − 0.08]	9%
Monteiro-Junior et al. (27) (R09)	General older adults	IL-6	SMD	5	− 0.81 [− 1.39, − 0.23]	90%
Hejazi et al. (23) (R05)	Multiple sclerosis	IL-6	SMD	10 arms	+ 0.09 [− 0.17, + 0.36]	92%
Sánchez-Romero et al. (33) (R15)	SASP older adults	TNF-alpha	MD	2	− 0.03 [− 1.44, + 1.38]	NR
Stigger et al. (34) (R16)	Dementia/MCI	TNF-alpha	MD	3	− 1.00 [− 1.90, − 0.10]	90%
Zanetti et al. (39) (R21)	HIV	TNF-alpha	MD	2	− 4.76 [− 10.81, + 1.29]	68%
Ubaghs et al. (36) (R18)	Breast cancer	TNF-alpha	SMD	4	− 0.05 [− 0.57, + 0.47]	78%
Malandish and Gulati et al. (25) (R07)	Heart failure/overweight-obesity	TNF-alpha	SMD	NR	− 0.06 [− 0.31, + 0.19]	75%
Khosravi et al. (24) (R06)	Cancer	TNF-alpha	SMD	NR	− 0.30 [− 0.53, − 0.06]	52%
Hejazi et al. (23) (R05)	Multiple sclerosis	TNF-alpha	SMD	9 arms	− 0.31 [− 0.56, − 0.06]	72%
Monteiro-Junior et al. (27) (R09)	General older adults	TNF-alpha	SMD	3	− 1.42 [− 2.96, + 0.13]	94%
Khosravi et al. (24) (R06)	Cancer	CRP	SMD	NR	− 0.47 [− 0.87, − 0.06]	67%
Ubaghs et al. (36) (R18)	Breast cancer	CRP	SMD	4	− 0.63 [− 1.51, + 0.25]	89%
Monteiro-Junior et al. (27) (R09)	General older adults	CRP	SMD	7	− 0.65 [− 1.11, − 0.18]	87%
Malandish and Gulati et al. (25) (R07)	Heart failure/overweight-obesity	hs-CRP	SMD	NR	− 0.38 [− 0.56, − 0.20]	74%
Sánchez-Romero et al. (33) (R15)	SASP older adults	IL-8	MD	2	− 0.28 [− 3.54, + 2.88]	NR
Khosravi et al. (24) (R06)	Cancer	IL-8	SMD	NR	− 0.30 [− 0.71, + 0.11]	64%
Malandish and Gulati et al. (25) (R07)	Heart failure/overweight-obesity	IL-8	SMD	NR	− 0.30 [− 0.74, + 0.14]	59%
Malandish and Gulati et al. (25) (R07)	Heart failure/overweight-obesity	IL-1beta	SMD	NR	+ 0.05 [− 0.32, + 0.43]	56%
Sánchez-Romero et al. (33) (R15)	SASP older adults	CCL2/MCP-1	MD	2	− 3.64 [− 25.91, + 15.59]	NR
Khosravi et al. (24) (R06)	Cancer	CCL2/MCP-1	SMD	NR	− 0.38 [− 0.79, + 0.02]	0%

Each row represents one systematic review’s own pooled estimate for a biomarker construct (exercise/physical activity vs. usual care, no exercise, or control). Estimates are reproduced from source reviews and are not repooled across reviews. SMD and MD metrics are not combined, populations are heterogeneous, and primary-study overlap is very high within the multiple sclerosis cluster. MD units were not reported consistently by the source reviews; MD estimates are therefore shown on their source scales and should not be compared across reviews. I² values are reported to the nearest whole per cent. A negative estimate favours exercise. Only core-eligible reviews are shown; evidence-map-only and narrative-only results, including Baker et al. (19) (R01), are provided in the **Supplementary Materials**.

*CI*, confidence interval; *CrI*, credible interval; *95% interval*, confidence or credible interval as reported by the source review; *CRP*, C-reactive protein; *hs-CRP*, high-sensitivity CRP; *IL*, interleukin; *k*, number of contributing primary studies or arms as reported; *MD*, mean difference; *SMD*, standardised mean difference; *TNF-alpha*, tumour necrosis factor alpha; *MCP-1*, monocyte chemoattractant protein-1; *NR*, not reported; *I^2^*, proportion of variability across primary studies attributable to heterogeneity.

IL-6. Five core-eligible reviews reported SMDs for IL-6, with point estimates ranging from − 0.81 (general older adults; Monteiro-Junior et al. (27), 95% CI: − 1.39 to − 0.23) to + 0.09 (multiple sclerosis; Hejazi et al. (23), − 0.17 to + 0.36). Two of the five confidence intervals excluded the null (Monteiro-Junior et al. (27); Malandish and Gulati et al. (25), − 0.20 [− 0.33, − 0.08]); the remainder were compatible with no effect, and within-review heterogeneity was often high (*I*^2^ up to 92%). Three reviews reported IL-6 as a mean difference and were directionally consistent with a reduction, most clearly in dementia/mild cognitive impairment (Stigger et al. (34), − 0.71 [− 0.88, − 0.54]); the Bayesian and HIV reviews had wide intervals crossing the null (R15, R21).

#### TNF-α

Five core-eligible reviews reported SMDs for TNF-α, from − 1.42 (Monteiro-Junior et al. (27), − 2.96 to + 0.13) to − 0.05 (breast cancer; Ubaghs et al. (36), − 0.57 to + 0.47). Confidence intervals excluded the null in two reviews (multiple sclerosis; Hejazi et al. (23), − 0.31 [− 0.56, − 0.06]; cancer; Khosravi et al. (24), − 0.30 [− 0.53, − 0.06]). Mean-difference reviews also suggested reductions, but intervals were wide except in dementia/mild cognitive impairment (Stigger et al. (34), − 1.00 [− 1.90, − 0.10]).

#### CRP

Three core-eligible reviews reported SMDs for CRP, all negative (− 0.65 [− 1.11, − 0.18] in general older adults (27), − 0.63 [− 1.51, + 0.25] in breast cancer (36), and − 0.47 [− 0.87, − 0.06] in cancer (24)); two of the three intervals excluded the null. One review reported hs-CRP with a reduction (heart failure with overweight/obesity; Malandish and Gulati et al. (25), − 0.38 [− 0.56, − 0.20]).

Overall, point estimates most consistently indicated small reductions in TNF-α and CRP, whereas IL-6 estimates were less consistent. Reviews reporting mean differences (summarised in **Table 2** and not plotted in **Figure 3**, which is restricted to a single metric) were directionally concordant. Statistical heterogeneity reported within source reviews was frequently substantial.

### Methodological quality of the reviews

Methodological quality was low across the evidence base (**Figure 4**; **Table 3**). Overall AMSTAR-2 confidence was critically low in 11 reviews, low in nine, and moderate in one (33); no review reached high confidence. The most frequent shortfalls were incomplete reporting of primary-study funding (item 10, 21 of 21 reviews) and the absence of a list of excluded studies with justification (item 7, 20 of 21), the latter being a critical domain; the absence of a registered or accessible protocol (item 2) was rated “no” in 10 reviews. Publication bias (item 15) was not assessable in nine reviews that performed no meta-analysis. Item-level ratings for every review are shown in **Figure 4**. With 20 of 21 reviews rated as having low or critically low confidence, all findings are interpreted with caution.

**Figure 4:**
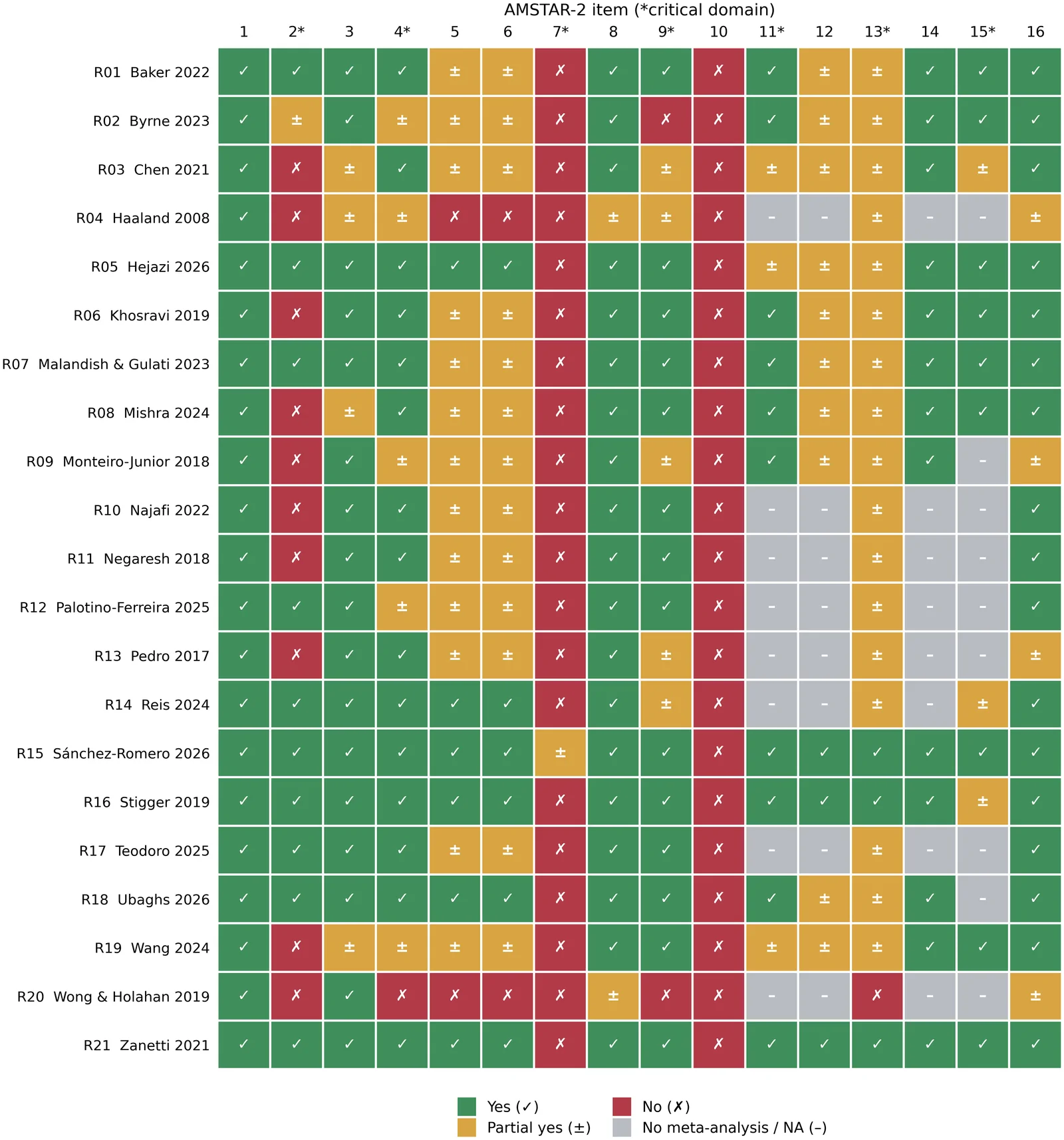
AMSTAR-2 item-level assessments across the included systematic reviews. AMSTAR-2 item-level assessment of the 21 included systematic reviews (rows) across items 1–16 (columns). Cells are colour- and symbol-coded so they remain interpretable without colour: ✓ yes; ± partial yes; ✗ no; – no meta-analysis or not applicable. Asterisks (*) denote AMSTAR-2 critical domains (items 2, 4, 7, 9, 11, 13, and 15). Overall confidence was critically low in 11 reviews, low in nine, and moderate in one. AMSTAR-2, A MeaSurement Tool to Assess systematic Reviews, version 2.

**Table 3:** Methodological quality of the included reviews (AMSTAR-2) and overall confidence.

Review	Overall confidence	Principal AMSTAR-2 limitations	Interpretation
Baker et al. (19) (R01)	Low	I7 excluded-study list; I10 primary-study funding not reported	One critical flaw; conclusions may not be trustworthy.
Byrne et al. (20) (R02)	Critically low	I2 protocol not accessible/published; I4 limited search; I7 excluded-study list; I9 bespoke RoB/quality approach	More than one critical flaw; do not rely on the review for a comprehensive synthesis.
Chen et al. (21) (R03)	Critically low	I2 protocol; I7 excluded-study list; I9/I13 human evidence RoB limitations	More than one critical flaw; do not rely on the review for a comprehensive synthesis.
Haaland et al. (22) (R04)	Critically low	I2 protocol; I4 search comprehensiveness; I7 excluded-study list; qualitative synthesis limited assessment of publication bias	More than one critical flaw; do not rely on the review for a comprehensive synthesis.
Hejazi et al. (23) (R05)	Low	I7 excluded-study list; I11 high-heterogeneity synthesis concerns	One critical flaw; conclusions may not be trustworthy.
Khosravi et al. (24) (R06)	Critically low	I2 protocol; I7 excluded-study list; I10 primary-study funding	More than one critical flaw; do not rely on the review for a comprehensive synthesis.
Malandish and Gulati et al. (25) (R07)	Low	I7 excluded-study list; I10 primary-study funding not reported	One critical flaw; conclusions may not be trustworthy.
Mishra et al. (26) (R08)	Critically low	I2 protocol; I7 excluded-study list; I9 high RoB evidence base; I13 interpretation limited	More than one critical flaw; do not rely on the review for a comprehensive synthesis.
Monteiro-Junior et al. (27) (R09)	Critically low	I2 protocol; I7 excluded-study list; I9 RoB tool/reporting unclear	More than one critical flaw; do not rely on the review for a comprehensive synthesis.
Najafi (28) (R10)	Critically low	I2 protocol; I7 excluded-study list; narrative-only synthesis limited assessment of quantitative synthesis and publication bias	More than one critical flaw; do not rely on the review for a comprehensive synthesis.
Negaresh (29) (R11)	Critically low	I2 protocol; I7 excluded-study list; qualitative synthesis limited assessment of publication bias	More than one critical flaw; do not rely on the review for a comprehensive synthesis.
Palotino-Ferreira (30) (R12)	Low	I7 excluded-study list; I4 search details incomplete in extracted text	One critical flaw; conclusions may not be trustworthy.
Pedro (31) (R13)	Critically low	I2 protocol; I7 excluded-study list; qualitative synthesis limited assessment of publication bias	More than one critical flaw; do not rely on the review for a comprehensive synthesis.
Reis (32) (R14)	Low	I7 excluded-study list; I9/I13 source-review RoB not primary-study RoB	One critical flaw; conclusions may not be trustworthy.
Sánchez-Romero et al. (33) (R15)	Moderate	No critical domain failed; incomplete excluded-study detail and primary-study funding reporting were noncritical limitations	More than one non-critical weakness; conclusions likely trustworthy.
Stigger et al. (34) (R16)	Low	I7 excluded-study list; I10 primary-study funding not reported	One critical flaw; conclusions may not be trustworthy.
Teodoro et al. (35) (R17)	Low	I7 excluded-study list; qualitative synthesis limited assessment of publication bias	One critical flaw; conclusions may not be trustworthy.
Ubaghs et al. (36) (R18)	Low	I7 excluded-study list; publication bias was not assessable or reported for the available analyses	One critical flaw; conclusions may not be trustworthy.
Wang et al. (37) (R19)	Critically low	I2 protocol; I4 limited search; I7 excluded-study list; mixed preclinical/clinical evidence	More than one critical flaw; do not rely on the review for a comprehensive synthesis.
Wong and Holahan (38) (R20)	Critically low	I2 protocol; I4 search details; I7 excluded-study list; I9 RoB tool unclear	More than one critical flaw; do not rely on the review for a comprehensive synthesis.
Zanetti et al. (39) (R21)	Low	I7 excluded-study list; I10 primary-study funding not reported	One critical flaw; conclusions may not be trustworthy.

AMSTAR-2 critical domains are protocol registration before commencement (item 2), adequacy of the literature search (item 4), justification for excluded studies (item 7), risk-of-bias assessment of included studies (item 9), appropriateness of meta-analytical methods (item 11), consideration of risk of bias when interpreting results (item 13), and assessment of publication bias (item 15). The “Principal AMSTAR-2 limitations” column includes failed critical domains and selected non-critical weaknesses relevant to interpretation. This column is abbreviated; complete item-level ratings are given in **Figure 4**. Overall confidence follows the AMSTAR-2 decision rules and the complete item-level ratings shown in **Figure 4**; a critical domain was counted as a critical flaw only when the corresponding item was rated “No”. Of the 21 reviews, confidence was critically low in 11, low in nine, and moderate in one; no review reached high confidence. Overall confidence is rated high, moderate, low or critically low on the basis of weaknesses in critical domains.

*AMSTAR-2*, A MeaSurement Tool to Assess systematic Reviews, version 2.

### *Post hoc* restricted descriptive sensitivity analysis

As a *post hoc* descriptive sensitivity analysis, we repeated the summary after excluding the seven core-eligible reviews rated critically low on AMSTAR-2, leaving seven core-eligible reviews (one moderate and six low confidence; **Supplementary Table 5**). Among SMD estimates, IL-6 ranged from − 0.20 to + 0.09 and TNF-α from − 0.31 to − 0.05, with one of three intervals excluding the null for each biomarker. The single remaining CRP SMD was − 0.63 [− 1.51, + 0.25], whereas the hs-CRP estimate remained below the null (− 0.38 [− 0.56, − 0.20]). The largest SMD reductions among the three primary biomarkers (IL-6: − 0.81, TNF-α: − 1.42, and CRP: − 0.65, all from Monteiro-Junior et al. (27)) were absent after restriction. Mean-difference evidence remained mixed and imprecise apart from the dementia/mild-cognitive-impairment review. This analysis generated no pooled estimate, does not upgrade certainty, and should be interpreted as a *post hoc* robustness check rather than a definitive best-evidence synthesis.

### Certainty of the evidence

Certainty of evidence, summarised at the construct level, was very low for every biomarker (**Table 4**). For the most frequently reported constructs, the certainty range spanned very low to moderate (IL-6, TNF-α) or very low to low (CRP, hs-CRP, IL-8, CCL2/MCP-1), but the most conservative grade—reflecting the least certain contributing evidence—was very low in all cases. Downgrading was driven mainly by risk of bias in the underlying primary studies, unexplained statistical heterogeneity (inconsistency), and imprecision. Given the predominance of very-low certainty, the summarised effect estimates should be regarded as uncertain.

**Table 4:** Certainty of the evidence by biomarker construct (GRADE).

Outcome (construct)	Framework	Reviews (core-eligible)	Certainty range	GRADE	Frequent downgrade domains
IL-6	GRADE	20 (14)	Very low to moderate	⊕○○○ Very low	Risk of bias (*n* = 20 of 22); inconsistency (*n* = 15 of 22); indirectness (*n* = 6 of 22); imprecision (*n* = 19 of 22) assessment entries
TNF-alpha	GRADE	19 (13)	Very low to moderate	⊕○○○ Very low	Risk of bias (*n* = 19 of 21); inconsistency (*n* = 17 of 21); indirectness (*n* = 6 of 21); imprecision (*n* = 19 of 21) assessment entries
CRP	GRADE	11 (6)	Very low to low	⊕○○○ Very low	Risk of bias (*n* = 11 of 11); inconsistency (*n* = 9 of 11); indirectness (*n* = 5 of 11); imprecision (*n* = 9 of 11) assessment entries
hs-CRP	GRADE	2 (2)	Very low to low	⊕○○○ Very low	Risk of bias (*n* = 2 of 2); inconsistency (*n* = 2 of 2); imprecision (*n* = 1 of 2) assessment entries
IL-8	GRADE	3 (3)	Very low to low	⊕○○○ Very low	Risk of bias (*n* = 2 of 4); inconsistency (*n* = 3 of 4); imprecision (*n* = 4 of 4) assessment entries
IL-1beta	GRADE	2 (2)	Very low	⊕○○○ Very low	Risk of bias (*n* = 2 of 2); inconsistency (*n* = 2 of 2); imprecision (*n* = 2 of 2) assessment entries
CCL2/MCP-1	GRADE	2 (2)	Very low to low	⊕○○○ Very low	Risk of bias (*n* = 1 of 3); inconsistency (*n* = 1 of 3); imprecision (*n* = 3 of 3) assessment entries
IL-18	GRADE	2 (2)	Very low	⊕○○○ Very low	Risk of bias (*n* = 2 of 2); inconsistency (*n* = 2 of 2); imprecision (*n* = 2 of 2) assessment entries
p16INK4a	GRADE	1 (0)	Very low	⊕○○○ Very low	Risk of bias (*n* = 1 of 1); inconsistency (*n* = 1 of 1); indirectness (*n* = 1 of 1); imprecision (*n* = 1 of 1) assessment entries
p21Cip1	GRADE	1 (0)	Very low	⊕○○○ Very low	Risk of bias (*n* = 1 of 1); inconsistency (*n* = 1 of 1); indirectness (*n* = 1 of 1); imprecision (*n* = 1 of 1) assessment entries
TNF-alpha/soluble TNF receptors	GRADE	1 (1)	Very low	⊕○○○ Very low	Risk of bias (*n* = 1 of 1); inconsistency (*n* = 1 of 1); imprecision (*n* = 1 of 1) assessment entries

Certainty is summarised descriptively at the construct level across contributing reviews. Where reviews gave different grades for the same construct, the range and the most conservative grade are reported; this is not a statistical pooling of certainty ratings. “Reviews (core-eligible)” gives the total number of contributing reviews with the core-eligible number in parentheses. Senescence markers (p16INK4a, p21Cip1) derive only from evidence-map-only evidence and do not inform the main conclusions. Grades are dominated by very-low certainty, so effect estimates should be regarded as uncertain. Counts in the “Frequent downgrade domains” column are the number of review–construct assessment entries in which the domain was downgraded, out of the total number of assessment entries for that construct. A review contributes more than one entry when both a source-reported and a reviewer-applied certainty rating were available, so these denominators can exceed the number of contributing reviews shown in the “Reviews (core-eligible)” column. A domain was counted as downgraded when the entry recorded a downgrade (reviewer-applied ratings) or a moderate, serious, or very serious concern (source-reported ratings); domains not listed for a construct were not downgraded in any entry. Full review-level entries are given in **Supplementary Table 4**.

*GRADE*, Grading of Recommendations Assessment, Development, and Evaluation. Certainty is rated high (⊕⊕⊕⊕), moderate (⊕⊕⊕○), low (⊕⊕○○), or very low (⊕○○○).

### Broader evidence map and less-studied markers

Mapping all constructs across both tiers confirmed that evidence was concentrated on IL-6 (20 reviews), TNF-α (19) and CRP (11), with much sparser data for hs-CRP, IL-8, IL-1β, CCL2/MCP-1, and IL-18 (each two to three reviews) and only single-review, very-low-certainty evidence for p16INK4a and p21Cip1 (**Figure 5**). The densest signal was a small reduction in TNF-α and CRP; IL-6 was mixed; and immunosenescence/senescence outcomes were too sparse for conclusions. Evidence-map-only reviews were R01, R02, R03, R08, R12, R14, and R19. R01 was map-only because adult-only pooled estimates were unavailable, while R19 was the only review restricted to stand-alone whole-body vibration. Neither review contributed to **Table 2** or **Figure 3**.

**Figure 5:**
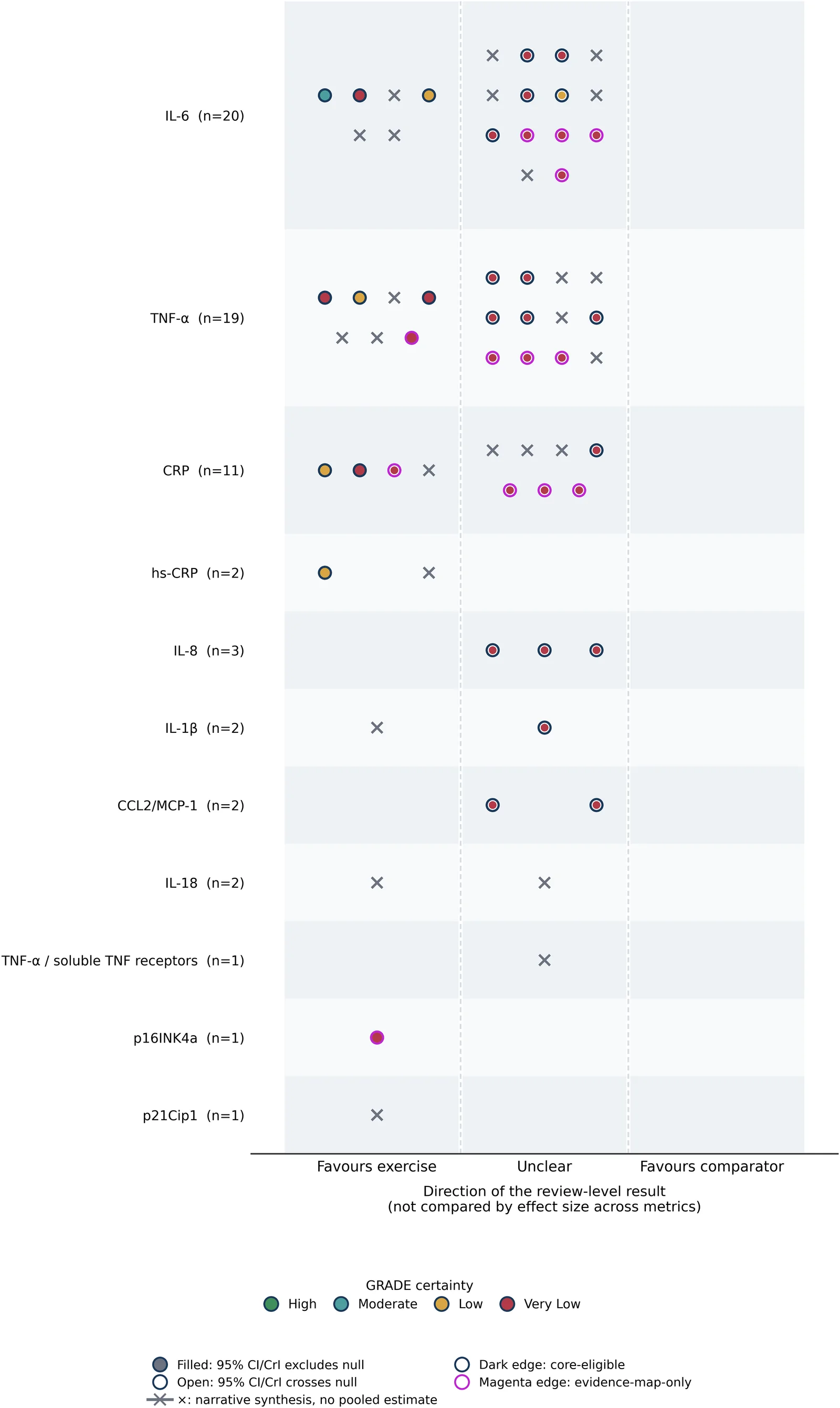
Evidence map of exercise effects on inflammaging and senescence biomarkers. Each marker represents one selected review-level result per review–biomarker construct. Overall analyses were preferred over subgroup or sensitivity analyses; when multiple nonnested comparisons remained, controlled between-group comparisons were preferred over within-person or cross-sectional comparisons. Columns show direction only (favours exercise, unclear, or favours comparator) and are not a magnitude scale because effect metrics differ. Filled markers denote 95% CI/CrI excluding the null, open markers denote intervals crossing the null, and crosses denote narrative syntheses without pooled estimates. Colour indicates GRADE certainty, and marker edge indicates evidence tier (dark, core-eligible; magenta, evidence-map-only). Marker counts should not be interpreted as independent confirmations or as a vote-counting synthesis because included reviews may share primary studies. CRP, C-reactive protein; CrI, credible interval; GRADE, Grading of Recommendations Assessment, Development, and Evaluation.

### Subgroup and sensitivity analyses reported within reviews

No umbrella-level meta-regression or subgroup analysis was prespecified or performed. Within a single multiple sclerosis review (23), exercise-modality subgroups were reported for IL-6 and TNF-α (**Supplementary Figure 1**; **Supplementary Table 7**): for TNF-α, high-intensity interval training (− 1.04 [− 1.71, − 0.37]) and aerobic training (− 0.56 [− 1.00, − 0.12]) showed reductions, whereas concurrent aerobic-plus-resistance training did not (+ 0.13 [− 0.27, + 0.53]); two subgroups were based on a single arm and therefore lacked a heterogeneity estimate (*I*^2^). These subgroups are hypothesis-generating and should not be read as robust effect modification. One sensitivity analysis (34) excluding the longest-duration study yielded a TNF-α mean difference of − 1.41 [− 1.89, − 0.93] with no heterogeneity. This sensitivity result and the full outcome-level evidence across all reviews and tiers are provided in **Supplementary Table 2**.

## Discussion

In this umbrella review of 21 systematic reviews, 14 core-eligible reviews contributed to the primary synthesis. Exercise and physical activity were associated with small reductions in some circulating inflammatory biomarkers—most consistently TNF-α and CRP—whereas effects on IL-6 were inconsistent and frequently close to the null. However, methodological confidence was low or critically low in 20 of 21 reviews, certainty was very low for every biomarker, statistical heterogeneity within source reviews was often high, and primary-study overlap was very high within the multiple sclerosis cluster. The evidence therefore points, at most, to modest anti-inflammatory effects of uncertain magnitude. In the *post hoc* restricted analysis, estimates moved closer to the null and the largest SMD reductions were no longer present, indicating that the apparent signal depends partly on reviews with critical methodological limitations.

The inconsistent IL-6 findings are biologically plausible because IL-6 has a dual role: it is released transiently from contracting muscle as a myokine, yet chronically elevated resting IL-6 is a feature of inflammaging and many chronic diseases (6, 8). Potential anti-inflammatory pathways include reductions in visceral adiposity and adipose-derived TNF-α, together with transient muscle-derived IL-6 signalling that can induce interleukin-10 and interleukin-1 receptor antagonist and suppress TNF-α production; CRP may decline downstream of reduced chronic IL-6 signalling (6–8). These are mechanistic proposals from exercise physiology. The small, heterogeneous and very-low-certainty biomarker changes summarised here cannot establish that these pathways operated at the exercise doses studied, and they are not the evidentiary basis of general physical-activity guidelines.

Our findings are consistent with the broader hypothesis that physical activity can attenuate aspects of inflammaging and immunosenescence (5, 9), but they also expose a clear gap: the intracellular senescence markers p16INK4a and p21Cip1 were addressed by only a single, very-low-certainty review (21), and the one review dedicated to senescence-associated secretory phenotype markers rested on only three randomised trials (33), even though senescence is central to the inflammaging construct (3, 4). Whether exercise acts as a senolytic or senomorphic intervention in humans therefore remains an open question that the current review-level evidence cannot answer.

Heterogeneity in this umbrella review has two distinct sources. Statistical heterogeneity was reported within source meta-analyses (*I*^2^ up to 94%) and reflects variation among primary trials in exercise dose and duration, assay and sampling protocol, and baseline inflammatory burden; it makes the magnitude of an individual pooled estimate unstable. Clinical diversity exists across the umbrella review: multiple sclerosis, cancer, HIV, heart failure, inflammatory bowel disease, dementia, frailty and healthy ageing differ in pathophysiology, treatment and age distribution. Consequently, a single transportable effect size for exercise on inflammation is not meaningful and was not estimated. The findings should be read as a review-level direction-of-effect pattern, not an effect applicable to a specific diagnosis or age stratum. Inference for a particular population should rest on the corresponding condition-specific review and its certainty rating.

Biological plausibility and comparatively definitive wording in source reviews should also be distinguished from clinical guidance. Monteiro-Junior et al. (27) concluded that exercise reduced IL-6 and CRP in older people, while explicitly acknowledging the small number of studies and methodological differences. Our appraisal rated that review critically low, and its comparatively large SMD estimates were absent from the restricted analysis. The cited World Health Organisation guideline does not recommend exercise specifically as an anti-inflammatory biomarker therapy; its recommendations to be physically active are based on broader clinical and functional outcomes (10). The narrower inference supported by the present review is therefore that the biomarker evidence alone is insufficient to justify an exercise prescription specifically for biomarker reduction.

### Strengths and limitations

Strengths of this umbrella review include comprehensive searching of six databases; transparent separation of core-eligible and evidence-map-only evidence; formal appraisal of methodological quality (AMSTAR-2), certainty (GRADE), and primary-study overlap (CCA); and prospective application of a separability rule to mixed adult/paediatric reviews. Quantifying overlap matters because redundant reviews of the same trials can create an illusion of independent replication (11). Several limitations must be emphasised. First, most reviews were of low or critically low methodological confidence, and certainty was uniformly very low. Second, heterogeneous effect metrics, comparators and populations precluded valid umbrella-level pooling. Third, many biomarkers were examined, so chance findings and selective emphasis remain possible. Fourth, publication bias was often unassessed and could not be reevaluated at review level. Fifth, exercise dose, adherence, assay methods, and sampling timing were incompletely reported. Sixth, the population spans older adults and several disease-defined adult groups, so no age-stratified or diagnosis-specific conclusion can be drawn from the umbrella-level ranges. Finally, the restricted analysis was *post hoc*, descriptive and unweighted; it is a robustness check, not a prespecified inferential analysis.

### Implications for research and practice

For clinical practice, the evidence is compatible with, but does not establish, an anti-inflammatory biomarker benefit of exercise; general recommendations to be physically active in older age and chronic disease rest on a much broader evidence base (10). Future randomised trials should prespecify a coherent biomarker panel and standardise measurement. A practical core would include hs-CRP, IL-6, TNF-α with soluble TNF receptors, IL-1β, IL-8/CXCL8, IL-10, and CCL2/MCP-1, supplemented by p16INK4a and p21Cip1 expression, leukocyte telomere length or telomerase activity, and immunosenescence phenotyping such as the CD4:CD8 ratio, naive and terminally differentiated effector-memory T-cell subsets, and CD28-negative/CD57-positive T cells. Sampling should be standardised for time of day, fasting state, and time since the last exercise bout, and trials should report assay platform, detection limits, exercise dose, and adherence. At the review level, priorities are prospective registration, complete excluded-study lists, explicit handling of overlap, and fewer redundant syntheses.

## Conclusion

Exercise and physical activity may produce small reductions in some circulating inflammatory biomarkers—most consistently TNF-α and CRP—in older adults and adults with chronic diseases, whereas effects on IL-6 are inconsistent. This evidence is of very low certainty and is drawn from reviews of predominantly low or critically low methodological confidence, with substantial primary-study overlap in some clusters. Effects on cellular-senescence and immunosenescence markers remain an evidence gap. The present biomarker evidence alone is insufficient to justify prescribing exercise specifically to lower inflammatory biomarkers; this conclusion does not alter the broader case for physical activity based on established clinical and functional benefits. Higher-quality primary trials and non-redundant, rigorously conducted syntheses are required.
