# Supplementary material for: Effects of exercise and physical activity on circulating inflammaging- and immunosenescence-related biomarkers in older adults and adults with chronic diseases: an umbrella review
Source: Front Immunol. 2026 Sep 29;17:1931607. doi: 10.3389/fimmu.2026.1931607 (PMC13659453; doi:10.3389/fimmu.2026.1931607)
Supplement: Supplementary file 1 [file DataSheet1.docx]

**Supplementary Materials**

**Supplementary Tables**

**Table S1. Database search strategy.**

| **Database** | **Search string (concept blocks combined with Boolean AND)** |
| --- | --- |
| #1 PubMed | #1 Population: "Aged"[Mesh] OR "Aging"[Mesh] OR "Chronic Disease"[Mesh] OR "Inflammatory Bowel Diseases"[Mesh] OR "Neoplasms"[Mesh] OR "HIV Infections"[Mesh] OR "Multiple Sclerosis"[Mesh] OR "Frailty"[Mesh] OR "Sarcopenia"[Mesh] OR "Obesity"[Mesh] OR "Heart Failure"[Mesh] OR "Dementia"[Mesh] OR "Cognitive Dysfunction"[Mesh] OR ("older adult*"[tiab] OR elderly[tiab] OR aged[tiab] OR geriatric*[tiab] OR "chronic disease*"[tiab] OR "inflammatory bowel disease*"[tiab] OR cancer*[tiab] OR neoplasm*[tiab] OR HIV[tiab] OR "multiple sclerosis"[tiab] OR frail*[tiab] OR sarcopeni*[tiab] OR obes*[tiab] OR overweight[tiab] OR "heart failure"[tiab] OR dementia[tiab] OR "cognitive impairment"[tiab] OR "mild cognitive impairment"[tiab]) #2 Intervention: "Exercise"[Mesh] OR "Exercise Therapy"[Mesh] OR "Resistance Training"[Mesh] OR "Walking"[Mesh] OR "Yoga"[Mesh] OR "Physical Fitness"[Mesh] OR "Vibration"[Mesh] OR (exercise*[tiab] OR "physical activity"[tiab] OR "physical training"[tiab] OR aerobic*[tiab] OR "resistance training"[tiab] OR "strength training"[tiab] OR "endurance training"[tiab] OR walking[tiab] OR "exercise rehabilitation"[tiab] OR "whole body vibration"[tiab] OR "vibration training"[tiab] OR yoga[tiab] OR "physical fitness"[tiab] OR "exercise therap*"[tiab]) #3 Outcome: "Interleukin-6"[Mesh] OR "Tumor Necrosis Factor-alpha"[Mesh] OR "C-Reactive Protein"[Mesh] OR "Interleukin-1beta"[Mesh] OR "Interleukin-8"[Mesh] OR "Chemokine CCL2"[Mesh] OR "Inflammation"[Mesh] OR "Cellular Senescence"[Mesh] OR "Cytokines"[Mesh] OR "Telomere"[Mesh] OR ("interleukin-6"[tiab] OR "IL-6"[tiab] OR "tumo?r necrosis factor*"[tiab] OR "TNF-alpha"[tiab] OR "TNF-a"[tiab] OR "C-reactive protein"[tiab] OR CRP[tiab] OR "hs-CRP"[tiab] OR "interleukin-1 beta"[tiab] OR "IL-1beta"[tiab] OR "interleukin-8"[tiab] OR "IL-8"[tiab] OR CXCL8[tiab] OR CCL2[tiab] OR "MCP-1"[tiab] OR "monocyte chemoattractant protein*"[tiab] OR inflammat*[tiab] OR inflammaging[tiab] OR cytokine*[tiab] OR "cellular senescence"[tiab] OR senescen*[tiab] OR p16[tiab] OR p16INK4a[tiab] OR p21[tiab] OR SASP[tiab] OR "senescence-associated secretory phenotype"[tiab] OR telomere*[tiab] OR telomerase[tiab] OR immunosenescence[tiab]) #4 Study design: "Systematic Review"[Publication Type] OR "Meta-Analysis"[Publication Type] OR ("systematic review*"[tiab] OR "meta-analys*"[tiab] OR "meta analys*"[tiab] OR "umbrella review*"[tiab] OR "overview of review*"[tiab]) #5 Combine: #1 AND #2 AND #3 AND #4 Filters: Humans[Mesh]; English. Search date: through 1 March 2026. |
| #2 Web of Science | #1 TS=("older adult*" OR elderly OR aged OR geriatric* OR "chronic disease*" OR "inflammatory bowel disease*" OR cancer* OR neoplasm* OR HIV OR "multiple sclerosis" OR frail* OR sarcopeni* OR obes* OR overweight OR "heart failure" OR dementia OR "cognitive impairment" OR "mild cognitive impairment") #2 TS=(exercise* OR "physical activity" OR "physical training" OR aerobic* OR "resistance training" OR "strength training" OR "endurance training" OR walking OR "exercise rehabilitation" OR "whole body vibration" OR "vibration training" OR yoga OR "physical fitness" OR "exercise therap*") #3 TS=("interleukin-6" OR "IL-6" OR "tumo?r necrosis factor*" OR "TNF-alpha" OR "TNF-a" OR "C-reactive protein" OR CRP OR "hs-CRP" OR "interleukin-1 beta" OR "IL-1beta" OR "interleukin-8" OR "IL-8" OR CXCL8 OR CCL2 OR "MCP-1" OR "monocyte chemoattractant protein*" OR inflammat* OR inflammaging OR cytokine* OR "cellular senescence" OR senescen* OR p16 OR p16INK4a OR p21 OR SASP OR "senescence-associated secretory phenotype" OR telomere* OR telomerase OR immunosenescence) #4 TS=("systematic review*" OR "meta-analys*" OR "meta analys*" OR "umbrella review*" OR "overview of review*") #5 #1 AND #2 AND #3 AND #4 Refine: Document Types = (Review OR Meta-Analysis); Language = English. Timespan: through 1 March 2026 (WoS operator wildcards * and ? used). |
| #3 Embase | #1 'aged'/exp OR 'chronic disease'/exp OR 'inflammatory bowel disease'/exp OR 'neoplasm'/exp OR 'human immunodeficiency virus infection'/exp OR 'multiple sclerosis'/exp OR 'frailty'/exp OR 'sarcopenia'/exp OR 'obesity'/exp OR 'heart failure'/exp OR 'dementia'/exp OR 'mild cognitive impairment'/exp OR ("older adult*":ti,ab,kw OR elderly:ti,ab,kw OR aged:ti,ab,kw OR geriatric*:ti,ab,kw OR "chronic disease*":ti,ab,kw OR "inflammatory bowel disease*":ti,ab,kw OR cancer*:ti,ab,kw OR neoplasm*:ti,ab,kw OR HIV:ti,ab,kw OR "multiple sclerosis":ti,ab,kw OR frail*:ti,ab,kw OR sarcopeni*:ti,ab,kw OR obes*:ti,ab,kw OR overweight:ti,ab,kw OR "heart failure":ti,ab,kw OR dementia:ti,ab,kw OR "cognitive impairment":ti,ab,kw OR "mild cognitive impairment":ti,ab,kw) #2 'exercise'/exp OR 'kinesiotherapy'/exp OR 'resistance training'/exp OR 'walking'/exp OR 'yoga'/exp OR 'whole body vibration'/exp OR 'fitness'/exp OR (exercise*:ti,ab,kw OR "physical activity":ti,ab,kw OR "physical training":ti,ab,kw OR aerobic*:ti,ab,kw OR "resistance training":ti,ab,kw OR "strength training":ti,ab,kw OR "endurance training":ti,ab,kw OR walking:ti,ab,kw OR "exercise rehabilitation":ti,ab,kw OR "whole body vibration":ti,ab,kw OR "vibration training":ti,ab,kw OR yoga:ti,ab,kw OR "physical fitness":ti,ab,kw OR "exercise therap*":ti,ab,kw) #3 'interleukin 6'/exp OR 'tumor necrosis factor'/exp OR 'C reactive protein'/exp OR 'interleukin 1beta'/exp OR 'interleukin 8'/exp OR 'monocyte chemotactic protein 1'/exp OR 'inflammation'/exp OR 'cell aging'/exp OR 'cytokine'/exp OR 'telomere'/exp OR ("interleukin-6":ti,ab,kw OR "IL-6":ti,ab,kw OR "tumo?r necrosis factor*":ti,ab,kw OR "TNF-alpha":ti,ab,kw OR "TNF-a":ti,ab,kw OR "C-reactive protein":ti,ab,kw OR CRP:ti,ab,kw OR "hs-CRP":ti,ab,kw OR "interleukin-1 beta":ti,ab,kw OR "IL-1beta":ti,ab,kw OR "interleukin-8":ti,ab,kw OR "IL-8":ti,ab,kw OR CXCL8:ti,ab,kw OR CCL2:ti,ab,kw OR "MCP-1":ti,ab,kw OR "monocyte chemoattractant protein*":ti,ab,kw OR inflammat*:ti,ab,kw OR inflammaging:ti,ab,kw OR cytokine*:ti,ab,kw OR "cellular senescence":ti,ab,kw OR senescen*:ti,ab,kw OR p16:ti,ab,kw OR p16INK4a:ti,ab,kw OR p21:ti,ab,kw OR SASP:ti,ab,kw OR "senescence-associated secretory phenotype":ti,ab,kw OR telomere*:ti,ab,kw OR telomerase:ti,ab,kw OR immunosenescence:ti,ab,kw) #4 'systematic review'/exp OR 'meta analysis'/exp OR ("systematic review*":ti,ab,kw OR "meta-analys*":ti,ab,kw OR "meta analys*":ti,ab,kw OR "umbrella review*":ti,ab,kw OR "overview of review*":ti,ab,kw) #5 #1 AND #2 AND #3 AND #4 Limits: [humans]/lim AND [english]/lim AND [embase]/lim. Search date: through 1 March 2026. |
| #4 Cochrane Library | #1 MeSH descriptor: [Aged] explode all trees OR [Chronic Disease] explode all trees OR [Neoplasms] explode all trees OR [Multiple Sclerosis] explode all trees OR [Frailty] OR [Sarcopenia] OR [Obesity] OR [Heart Failure] OR [Dementia] OR [Cognitive Dysfunction] OR ("older adult*":ti,ab,kw OR elderly:ti,ab,kw OR aged:ti,ab,kw OR geriatric*:ti,ab,kw OR "chronic disease*":ti,ab,kw OR "inflammatory bowel disease*":ti,ab,kw OR cancer*:ti,ab,kw OR neoplasm*:ti,ab,kw OR HIV:ti,ab,kw OR "multiple sclerosis":ti,ab,kw OR frail*:ti,ab,kw OR sarcopeni*:ti,ab,kw OR obes*:ti,ab,kw OR overweight:ti,ab,kw OR "heart failure":ti,ab,kw OR dementia:ti,ab,kw OR "cognitive impairment":ti,ab,kw OR "mild cognitive impairment":ti,ab,kw) #2 MeSH descriptor: [Exercise] explode all trees OR [Exercise Therapy] explode all trees OR [Resistance Training] OR [Walking] OR [Yoga] OR [Vibration] OR (exercise*:ti,ab,kw OR "physical activity":ti,ab,kw OR "physical training":ti,ab,kw OR aerobic*:ti,ab,kw OR "resistance training":ti,ab,kw OR "strength training":ti,ab,kw OR "endurance training":ti,ab,kw OR walking:ti,ab,kw OR "exercise rehabilitation":ti,ab,kw OR "whole body vibration":ti,ab,kw OR "vibration training":ti,ab,kw OR yoga:ti,ab,kw OR "physical fitness":ti,ab,kw OR "exercise therap*":ti,ab,kw) #3 MeSH descriptor: [Interleukin-6] OR [Tumor Necrosis Factor-alpha] OR [C-Reactive Protein] OR [Interleukin-1beta] OR [Interleukin-8] OR [Chemokine CCL2] OR [Inflammation] OR [Cellular Senescence] OR [Telomere] OR ("interleukin-6":ti,ab,kw OR "IL-6":ti,ab,kw OR "tumo?r necrosis factor*":ti,ab,kw OR "TNF-alpha":ti,ab,kw OR "TNF-a":ti,ab,kw OR "C-reactive protein":ti,ab,kw OR CRP:ti,ab,kw OR "hs-CRP":ti,ab,kw OR "interleukin-1 beta":ti,ab,kw OR "IL-1beta":ti,ab,kw OR "interleukin-8":ti,ab,kw OR "IL-8":ti,ab,kw OR CXCL8:ti,ab,kw OR CCL2:ti,ab,kw OR "MCP-1":ti,ab,kw OR "monocyte chemoattractant protein*":ti,ab,kw OR inflammat*:ti,ab,kw OR inflammaging:ti,ab,kw OR cytokine*:ti,ab,kw OR "cellular senescence":ti,ab,kw OR senescen*:ti,ab,kw OR p16:ti,ab,kw OR p16INK4a:ti,ab,kw OR p21:ti,ab,kw OR SASP:ti,ab,kw OR "senescence-associated secretory phenotype":ti,ab,kw OR telomere*:ti,ab,kw OR telomerase:ti,ab,kw OR immunosenescence:ti,ab,kw) #4 ("systematic review*":ti,ab,kw OR "meta-analys*":ti,ab,kw OR "meta analys*":ti,ab,kw OR "umbrella review*":ti,ab,kw OR "overview of review*":ti,ab,kw) #5 #1 AND #2 AND #3 AND #4 Note: run in CDSR for systematic reviews. Search date: through 1 March 2026. |
| #5 Scopus | #1 TITLE-ABS-KEY("older adult*" OR elderly OR aged OR geriatric* OR "chronic disease*" OR "inflammatory bowel disease*" OR cancer* OR neoplasm* OR HIV OR "multiple sclerosis" OR frail* OR sarcopeni* OR obes* OR overweight OR "heart failure" OR dementia OR "cognitive impairment" OR "mild cognitive impairment") #2 TITLE-ABS-KEY(exercise* OR "physical activity" OR "physical training" OR aerobic* OR "resistance training" OR "strength training" OR "endurance training" OR walking OR "exercise rehabilitation" OR "whole body vibration" OR "vibration training" OR yoga OR "physical fitness" OR "exercise therap*") #3 TITLE-ABS-KEY("interleukin-6" OR "IL-6" OR "tumo?r necrosis factor*" OR "TNF-alpha" OR "TNF-a" OR "C-reactive protein" OR CRP OR "hs-CRP" OR "interleukin-1 beta" OR "IL-1beta" OR "interleukin-8" OR "IL-8" OR CXCL8 OR CCL2 OR "MCP-1" OR "monocyte chemoattractant protein*" OR inflammat* OR inflammaging OR cytokine* OR "cellular senescence" OR senescen* OR p16 OR p16INK4a OR p21 OR SASP OR "senescence-associated secretory phenotype" OR telomere* OR telomerase OR immunosenescence) #4 TITLE-ABS-KEY("systematic review*" OR "meta-analys*" OR "meta analys*" OR "umbrella review*" OR "overview of review*") #5 #1 AND #2 AND #3 AND #4 Limit: LIMIT-TO(DOCTYPE,"re") AND LIMIT-TO(LANGUAGE,"English"). Search date: through 1 March 2026. |
| #6 SPORTDiscus | #1 (DE "OLDER people" OR DE "CHRONIC diseases" OR DE "MULTIPLE sclerosis" OR DE "FRAILTY" OR DE "SARCOPENIA" OR DE "OBESITY" OR DE "HEART failure" OR DE "DEMENTIA") OR TI("older adult*" OR elderly OR aged OR geriatric* OR "chronic disease*" OR "inflammatory bowel disease*" OR cancer* OR neoplasm* OR HIV OR "multiple sclerosis" OR frail* OR sarcopeni* OR obes* OR overweight OR "heart failure" OR dementia OR "cognitive impairment" OR "mild cognitive impairment") OR AB("older adult*" OR elderly OR aged OR geriatric* OR "chronic disease*" OR "inflammatory bowel disease*" OR cancer* OR neoplasm* OR HIV OR "multiple sclerosis" OR frail* OR sarcopeni* OR obes* OR overweight OR "heart failure" OR dementia OR "cognitive impairment" OR "mild cognitive impairment") #2 (DE "EXERCISE" OR DE "PHYSICAL activity" OR DE "RESISTANCE training" OR DE "AEROBIC exercises" OR DE "WALKING" OR DE "YOGA" OR DE "WHOLE body vibration") OR TI(exercise* OR "physical activity" OR "physical training" OR aerobic* OR "resistance training" OR "strength training" OR "endurance training" OR walking OR "exercise rehabilitation" OR "whole body vibration" OR "vibration training" OR yoga OR "physical fitness" OR "exercise therap*") OR AB(exercise* OR "physical activity" OR "physical training" OR aerobic* OR "resistance training" OR "strength training" OR "endurance training" OR walking OR "exercise rehabilitation" OR "whole body vibration" OR "vibration training" OR yoga OR "physical fitness" OR "exercise therap*") #3 (DE "INTERLEUKIN-6" OR DE "TUMOR necrosis factors" OR DE "C-reactive protein" OR DE "INFLAMMATION" OR DE "CELLULAR aging" OR DE "CYTOKINES" OR DE "TELOMERES") OR TI("interleukin-6" OR "IL-6" OR "tumo?r necrosis factor*" OR "TNF-alpha" OR "TNF-a" OR "C-reactive protein" OR CRP OR "hs-CRP" OR "interleukin-1 beta" OR "IL-1beta" OR "interleukin-8" OR "IL-8" OR CXCL8 OR CCL2 OR "MCP-1" OR "monocyte chemoattractant protein*" OR inflammat* OR inflammaging OR cytokine* OR "cellular senescence" OR senescen* OR p16 OR p16INK4a OR p21 OR SASP OR "senescence-associated secretory phenotype" OR telomere* OR telomerase OR immunosenescence) OR AB("interleukin-6" OR "IL-6" OR "tumo?r necrosis factor*" OR "TNF-alpha" OR "TNF-a" OR "C-reactive protein" OR CRP OR "hs-CRP" OR "interleukin-1 beta" OR "IL-1beta" OR "interleukin-8" OR "IL-8" OR CXCL8 OR CCL2 OR "MCP-1" OR "monocyte chemoattractant protein*" OR inflammat* OR inflammaging OR cytokine* OR "cellular senescence" OR senescen* OR p16 OR p16INK4a OR p21 OR SASP OR "senescence-associated secretory phenotype" OR telomere* OR telomerase OR immunosenescence) #4 TI("systematic review*" OR "meta-analys*" OR "meta analys*" OR "umbrella review*" OR "overview of review*") OR AB("systematic review*" OR "meta-analys*" OR "meta analys*" OR "umbrella review*" OR "overview of review*") #5 S1 AND S2 AND S3 AND S4 Limiters: Language = English. Search date: through 1 March 2026. |

Concept blocks: #1 population (older adults and adults with chronic disease); #2 intervention/exposure (exercise and physical activity, including aerobic, resistance, combined, walking, whole-body vibration and yoga); #3 outcome (circulating inflammatory/inflammaging biomarkers and senescence/immunosenescence markers); #4 study design (systematic reviews, meta-analyses, umbrella reviews). The four blocks were combined with the Boolean operator AND. Controlled vocabulary (MeSH/Emtree/subject headings) and free-text terms were both used; * and ? are truncation/wildcard operators.

Databases searched: PubMed, Web of Science Core Collection, Embase, Cochrane Library, Scopus and SPORTDiscus. All searches were run through 1 March 2026 and limited to human studies and English-language records where the interface allowed. The strings above reflect the searches as executed.

**Table S2. Outcome-level quantitative and narrative evidence reported across all included reviews.**

| **Review** | **Tier** | **Condition** | **Outcome** | **Comparator** | **Metric** | **Effect [95% interval]** | **I²** | **Synthesis role** |
| --- | --- | --- | --- | --- | --- | --- | --- | --- |
| Baker 2022 (R01) | Map | IBD | IL-6 | Usual care/no exercise/control | SMD | -0.09 [-0.49, +0.30] | 0% | Map only |
| Haaland 2008 (R04) | Core | Healthy older adults | IL-6 | Usual care/no exercise/control | Narrative | NR | NA | Narrative only |
| Hejazi 2026 (R05) | Core | Multiple sclerosis | IL-6 | Usual care/no exercise/control | SMD | +0.09 [-0.17, +0.36] | 92% | Primary |
| Hejazi 2026 (R05) | Core | Multiple sclerosis | IL-6 | Usual care/no exercise/control | SMD | +0.30 [-0.04, +0.63] | 94% | Subgroup |
| Hejazi 2026 (R05) | Core | Multiple sclerosis | IL-6 | Usual care/no exercise/control | SMD | -0.39 [-0.81, +0.04] | 51% | Subgroup |
| Khosravi 2019 (R06) | Core | Cancer | IL-6 | Usual care/no exercise/control | SMD | -0.16 [-0.38, +0.06] | 38% | Primary |
| Malandish & Gulati 2023 (R07) | Core | Heart failure/overweight-obesity | IL-6 | Usual care/no exercise/control | SMD | -0.20 [-0.33, -0.08] | 9% | Primary |
| Monteiro-Junior 2018 (R09) | Core | General older adults | IL-6 | Usual care/no exercise/control | SMD | -0.81 [-1.39, -0.23] | 90% | Primary |
| Najafi 2022 (R10) | Core | Multiple sclerosis | IL-6 | Usual care/no exercise/control | Narrative | NR | NA | Narrative only |
| Negaresh 2018 (R11) | Core | Multiple sclerosis | IL-6 | Usual care/no exercise/control | Narrative | NR | NA | Narrative only |
| Pedro 2017 (R13) | Core | HIV | IL-6 | Usual care/no exercise/control | Narrative | NR | NA | Narrative only |
| Sánchez-Romero 2026 (R15) | Core | SASP older adults | IL-6 | Usual care/no exercise/control | MD | -0.04 [-0.97, +0.91] | NR | Primary |
| Stigger 2019 (R16) | Core | Dementia/MCI | IL-6 | Usual care/no exercise/control | MD | -0.71 [-0.88, -0.54] | 0% | Primary |
| Teodoro 2025 (R17) | Core | Older adults TH1/TH2 | IL-6 | Usual care/no exercise/control | Narrative | NR | NA | Narrative only |
| Teodoro 2025 (R17) | Core | Older adults TH1/TH2 | IL-6 | Baseline/pre-intervention | Narrative | NR | NA | Narrative only |
| Ubaghs 2026 (R18) | Core | Breast cancer | IL-6 | Usual care/no exercise/control | SMD | -0.01 [-0.24, +0.21] | 27% | Primary |
| Wong & Holahan 2019 (R20) | Core | Multiple sclerosis | IL-6 | Usual care/no exercise/control | Narrative | NR | NA | Narrative only |
| Zanetti 2021 (R21) | Core | HIV | IL-6 | Usual care/no exercise/control | MD | -1.18 [-3.71, +1.35] | 93% | Primary |
| Baker 2022 (R01) | Map | IBD | TNF-alpha | Usual care/no exercise/control | SMD | +0.08 [-0.31, +0.48] | 0% | Map only |
| Hejazi 2026 (R05) | Core | Multiple sclerosis | TNF-alpha | Usual care/no exercise/control | SMD | -0.31 [-0.56, -0.06] | 72% | Primary |
| Hejazi 2026 (R05) | Core | Multiple sclerosis | TNF-alpha | Usual care/no exercise/control | SMD | -0.56 [-1.00, -0.12] | 76% | Subgroup |
| Hejazi 2026 (R05) | Core | Multiple sclerosis | TNF-alpha | Usual care/no exercise/control | SMD | +0.13 [-0.27, +0.53] | 60% | Subgroup |
| Hejazi 2026 (R05) | Core | Multiple sclerosis | TNF-alpha | Usual care/no exercise/control | SMD | -0.25 [-0.97, +0.47] | NA | Subgroup |
| Hejazi 2026 (R05) | Core | Multiple sclerosis | TNF-alpha | Usual care/no exercise/control | SMD | -1.04 [-1.71, -0.37] | NA | Subgroup |
| Khosravi 2019 (R06) | Core | Cancer | TNF-alpha | Usual care/no exercise/control | SMD | -0.30 [-0.53, -0.06] | 52% | Primary |
| Malandish & Gulati 2023 (R07) | Core | Heart failure/overweight-obesity | TNF-alpha | Usual care/no exercise/control | SMD | -0.06 [-0.31, +0.19] | 75% | Primary |
| Monteiro-Junior 2018 (R09) | Core | General older adults | TNF-alpha | Usual care/no exercise/control | SMD | -1.42 [-2.96, +0.13] | 94% | Primary |
| Najafi 2022 (R10) | Core | Multiple sclerosis | TNF-alpha | Usual care/no exercise/control | Narrative | NR | NA | Narrative only |
| Negaresh 2018 (R11) | Core | Multiple sclerosis | TNF-alpha | Usual care/no exercise/control | Narrative | NR | NA | Narrative only |
| Pedro 2017 (R13) | Core | HIV | TNF-alpha | Usual care/no exercise/control | Narrative | NR | NA | Narrative only |
| Sánchez-Romero 2026 (R15) | Core | SASP older adults | TNF-alpha | Usual care/no exercise/control | MD | -0.03 [-1.44, +1.38] | NR | Primary |
| Stigger 2019 (R16) | Core | Dementia/MCI | TNF-alpha | Usual care/no exercise/control | MD | -1.00 [-1.90, -0.10] | 90% | Primary |
| Stigger 2019 (R16) | Core | Dementia/MCI | TNF-alpha | Usual care/no exercise/control | MD | -1.41 [-1.89, -0.93] | 0% | Sensitivity |
| Teodoro 2025 (R17) | Core | Older adults TH1/TH2 | TNF-alpha | Usual care/no exercise/control | Narrative | NR | NA | Narrative only |
| Ubaghs 2026 (R18) | Core | Breast cancer | TNF-alpha | Usual care/no exercise/control | SMD | -0.05 [-0.57, +0.47] | 78% | Primary |
| Wong & Holahan 2019 (R20) | Core | Multiple sclerosis | TNF-alpha | Usual care/no exercise/control | Narrative | NR | NA | Narrative only |
| Zanetti 2021 (R21) | Core | HIV | TNF-alpha | Usual care/no exercise/control | MD | -4.76 [-10.81, +1.29] | 68% | Primary |
| Baker 2022 (R01) | Map | IBD | CRP | Usual care/no exercise/control | SMD | -0.04 [-0.58, +0.50] | 0% | Map only |
| Haaland 2008 (R04) | Core | Healthy older adults | CRP | Usual care/no exercise/control | Narrative | NR | NA | Narrative only |
| Khosravi 2019 (R06) | Core | Cancer | CRP | Usual care/no exercise/control | SMD | -0.47 [-0.87, -0.06] | 67% | Primary |
| Monteiro-Junior 2018 (R09) | Core | General older adults | CRP | Usual care/no exercise/control | SMD | -0.65 [-1.11, -0.18] | 87% | Primary |
| Najafi 2022 (R10) | Core | Multiple sclerosis | CRP | Usual care/no exercise/control | Narrative | NR | NA | Narrative only |
| Stigger 2019 (R16) | Core | Dementia/MCI | CRP | Usual care/no exercise/control | Narrative | NR | NA | Narrative only |
| Ubaghs 2026 (R18) | Core | Breast cancer | CRP | Usual care/no exercise/control | SMD | -0.63 [-1.51, +0.25] | 89% | Primary |
| Malandish & Gulati 2023 (R07) | Core | Heart failure/overweight-obesity | hs-CRP | Usual care/no exercise/control | SMD | -0.38 [-0.56, -0.20] | 74% | Primary |
| Pedro 2017 (R13) | Core | HIV | hs-CRP | Usual care/no exercise/control | Narrative | NR | NA | Narrative only |
| Khosravi 2019 (R06) | Core | Cancer | IL-8 | Usual care/no exercise/control | SMD | -0.30 [-0.71, +0.11] | 64% | Primary |
| Malandish & Gulati 2023 (R07) | Core | Heart failure/overweight-obesity | IL-8 | Usual care/no exercise/control | SMD | -0.30 [-0.74, +0.14] | 59% | Primary |
| Sánchez-Romero 2026 (R15) | Core | SASP older adults | IL-8 | Usual care/no exercise/control | MD | -0.28 [-3.54, +2.88] | NR | Primary |
| Malandish & Gulati 2023 (R07) | Core | Heart failure/overweight-obesity | IL-1beta | Usual care/no exercise/control | SMD | +0.05 [-0.32, +0.43] | 56% | Primary |
| Stigger 2019 (R16) | Core | Dementia/MCI | IL-1beta | Usual care/no exercise/control | Narrative | NR | NA | Narrative only |
| Khosravi 2019 (R06) | Core | Cancer | CCL2/MCP-1 | Usual care/no exercise/control | SMD | -0.38 [-0.79, +0.02] | 0% | Primary |
| Sánchez-Romero 2026 (R15) | Core | SASP older adults | CCL2/MCP-1 | Usual care/no exercise/control | MD | -3.64 [-25.91, +15.59] | NR | Primary |
| Haaland 2008 (R04) | Core | Healthy older adults | TNF-alpha / soluble TNF receptors | Usual care/no exercise/control | Narrative | NR | NA | Narrative only |
| Haaland 2008 (R04) | Core | Healthy older adults | IL-18 | Usual care/no exercise/control | Narrative | NR | NA | Narrative only |
| Pedro 2017 (R13) | Core | HIV | IL-18 | Usual care/no exercise/control | Narrative | NR | NA | Narrative only |
| Byrne 2023 (R02) | Map | Frailty/sarcopenia | IL-6 | Usual care/no exercise/control | SMD | -0.28 [-0.57, +0.00] | 46% | Map only |
| Mishra 2024 (R08) | Map | Yoga/mixed conditions | IL-6 | Usual care/no exercise/control | SMD | -0.33 [-0.80, +0.13] | 90% | Map only |
| Palotino-Ferreira 2025 (R12) | Map | Vaccination/physical activity | IL-6 | Usual care/no exercise/control | Narrative | NR | NA | Map only |
| Reis 2024 (R14) | Map | Nested overview | IL-6 | Comparator as reported | Narrative | NR | NA | Map only |
| Wang 2024 (R19) | Map | Whole-body vibration | IL-6 | Usual care/no exercise/control | SMD | +0.06 [-0.32, +0.45] | 64% | Map only |
| Wang 2024 (R19) | Map | Whole-body vibration | IL-6 | Baseline/pre-intervention | SMD | +0.91 [+0.31, +1.52] | 93% | Map only |
| Byrne 2023 (R02) | Map | Frailty/sarcopenia | TNF-alpha | Usual care/no exercise/control | SMD | -0.12 [-0.47, +0.22] | 67% | Map only |
| Mishra 2024 (R08) | Map | Yoga/mixed conditions | TNF-alpha | Usual care/no exercise/control | SMD | -0.29 [-0.59, +0.01] | 46% | Map only |
| Palotino-Ferreira 2025 (R12) | Map | Vaccination/physical activity | TNF-alpha | Usual care/no exercise/control | Narrative | NR | NA | Map only |
| Reis 2024 (R14) | Map | Nested overview | TNF-alpha | Comparator as reported | Narrative | NR | NA | Map only |
| Wang 2024 (R19) | Map | Whole-body vibration | TNF-alpha | Usual care/no exercise/control | SMD | -1.11 [-2.16, -0.06] | 91% | Map only |
| Wang 2024 (R19) | Map | Whole-body vibration | TNF-alpha | Baseline/pre-intervention | SMD | -0.02 [-1.31, +1.27] | 95% | Map only |
| Byrne 2023 (R02) | Map | Frailty/sarcopenia | CRP | Usual care/no exercise/control | SMD | -0.28 [-0.56, +0.00] | 69% | Map only |
| Mishra 2024 (R08) | Map | Yoga/mixed conditions | CRP | Usual care/no exercise/control | SMD | -0.15 [-0.89, +0.59] | 96% | Map only |
| Reis 2024 (R14) | Map | Nested overview | CRP | Comparator as reported | Narrative | NR | NA | Map only |
| Wang 2024 (R19) | Map | Whole-body vibration | CRP | Usual care/no exercise/control | SMD | -5.08 [-14.82, +4.66] | 99% | Map only |
| Wang 2024 (R19) | Map | Whole-body vibration | CRP | Baseline/pre-intervention | SMD | -1.81 [-3.97, +0.35] | 98% | Map only |
| Wang 2024 (R19) | Map | Whole-body vibration | CRP | Baseline/pre-intervention | SMD | -3.59 [-6.36, -0.82] | 98% | Map only |
| Chen 2021 (R03) | Map | Senolytic/aging human | p16INK4a | Lower activity/reference | Correlation coefficient (r) | -0.30 [-0.54, +0.01] | 84% | Map only |
| Chen 2021 (R03) | Map | Senolytic/aging human | p16INK4a | Baseline/pre-intervention | Relative change (%) | -64% [-72%, -55%] | 0% | Map only |
| Chen 2021 (R03) | Map | Senolytic/aging human | p21Cip1 | Usual care/no exercise/control | Narrative | NR | NA | Map only |

CI, confidence interval; CrI, credible interval; CRP, C-reactive protein; IL, interleukin; MCP-1, monocyte chemoattractant protein-1; MD, mean difference; SMD, standardized mean difference; TNF-alpha, tumour necrosis factor alpha; I², heterogeneity statistic; 95% interval, confidence or credible interval as reported by the source review; NR, not reported by the source review; NA, not applicable or not estimable.

Tier: Core = core-eligible; Map = evidence-map-only. Synthesis role identifies whether a row contributes to the primary synthesis or is retained as a source-reported subgroup, sensitivity, narrative-only, or evidence-map-only result. Baker 2022 (R01) is Map because the source meta-analysis included mixed adult/paediatric evidence without separable adult-only pooled estimates. For narrative-only findings, no numerical effect estimate or interval was available; the Effect cell is therefore NR and I² is NA. I² is NR when it was not reported for a quantitative estimate and NA when it was not estimable, including single-arm subgroups. A negative estimate favours exercise. Estimates are reproduced from source reviews and are not re-pooled across reviews.I² values are reported to the nearest whole percent.

**Table S3. Age criteria and reported participant age of the included reviews.**

| **Review** | **Condition / population** | **Age criterion stated by the source review** | **Participant age as reported** | **Age category in this umbrella review** |
| --- | --- | --- | --- | --- |
| Baker 2022 (R01) | IBD | No numeric age criterion (adults with IBD) | Most included trials enrolled participants aged 16 years and over, several aged 18–40 years; one paediatric trial (mean 14.5 years) | Mixed adult/paediatric (evidence-map-only) |
| Byrne 2023 (R02) | Frailty/sarcopenia | Adults aged ≥60 years | Pre-frail, frail or sarcopenic adults aged ≥60 years | Older adults |
| Chen 2021 (R03) | Senolytic/ageing biology | No numeric age criterion (human studies) | Participants aged 18–81 years across the included studies | Mixed adult-to-older |
| Haaland 2008 (R04) | Healthy older adults | Older populations, all participants aged at least 50 years | Mean age reported for each included trial | Older adults (≥50 years) |
| Hejazi 2026 (R05) | Multiple sclerosis | Patients aged ≥18 years with multiple sclerosis | Mean age across the 19 trials 27–50 years | Adults with chronic disease |
| Khosravi 2019 (R06) | Cancer survivors | Adult cancer survivors, no numeric threshold | Mean age reported per trial (e.g. 65.7 and 67.9 years in the exercise and control arms of one trial) | Mixed adult-to-older |
| Malandish & Gulati 2023 (R07) | Heart failure with overweight/obesity | No numeric age criterion | Patients aged 49–77 years | Mixed adult-to-older |
| Mishra 2024 (R08) | Yoga, mixed conditions | No numeric age criterion | Age undefined in several included trials; remaining trials in mid-adulthood | Mixed adult |
| Monteiro-Junior 2018 (R09) | General older adults | Older people aged ≥60 years | ≥60 years by inclusion criterion | Older adults |
| Najafi 2022 (R10) | Multiple sclerosis | No numeric age criterion | Mean age reported per trial, approximately 25–54 years | Adults with chronic disease |
| Negaresh 2018 (R11) | Multiple sclerosis | No numeric age criterion | Participants aged 18–60 years | Adults with chronic disease |
| Palotino-Ferreira 2025 (R12) | Vaccination and physical activity | Adults or older adults, no numeric threshold | Included studies ranged from young adults to older adults | Mixed adult-to-older |
| Pedro 2017 (R13) | HIV | Participants aged 18–60 years | 18–60 years by inclusion criterion | Adults with chronic disease |
| Reis 2024 (R14) | Nested overview of reviews | No numeric age criterion | Age range 17–95 years across the reviews analysed | Mixed adult-to-older |
| Sánchez-Romero 2026 (R15) | SASP older adults | Participants aged ≥60 years | Age range 66–79 years | Older adults |
| Stigger 2019 (R16) | Dementia/MCI | Mean age ≥60 years | Mean age ≥60 years by inclusion criterion | Older adults |
| Teodoro 2025 (R17) | Older adults (TH1/TH2) | Mean age ≥60 years | Mean age ≥60 years by inclusion criterion | Older adults |
| Ubaghs 2026 (R18) | Breast cancer | Women aged ≥18 years in most included trials | Trial-level criteria ranged from ≥18 years to 20–65 years | Adults with chronic disease |
| Wang 2024 (R19) | Whole-body vibration | No numeric age criterion; mixed preclinical and clinical evidence | Clinical subset conducted in older adults | Mixed (evidence-map-only) |
| Wong & Holahan 2019 (R20) | Multiple sclerosis | No numeric age criterion | NR at review level | Adults with chronic disease |
| Zanetti 2021 (R21) | HIV | People living with HIV aged 18–60 years | Mean age 41.0 years in the five trials reporting it | Adults with chronic disease |

IBD, inflammatory bowel disease; MCI, mild cognitive impairment; NR, not reported; SASP, senescence-associated secretory phenotype.

Entries are reproduced from each source review. Older adults indicates a source review requiring participants aged ≥60 years or a mean age ≥60 years (or ≥50 years where explicitly defined); adults with chronic disease indicates a disease-defined adult population without an older-age threshold; mixed adult-to-older indicates a review spanning both. Baker 2022 included a paediatric Crohn disease trial and was therefore reclassified as evidence-map-only because adult-only pooled estimates were unavailable. Age heterogeneity is addressed as a limitation on generalisability.

**Table S4. Certainty of evidence (GRADE) by review and biomarker construct.**

| **Review** | **Outcome** | **Source** | **Certainty** | **Downgrade considerations** | **Tier** |
| --- | --- | --- | --- | --- | --- |
| Baker 2022 (R01) | IL-6 | Reviewer-applied | Very Low | Risk of bias=-1; Inconsistency=No/unclear; Indirectness=-1 (mixed adult/paediatric evidence); Imprecision=-1; Pub. bias: none | Map |
| Byrne 2023 (R02) | IL-6 | Reviewer-applied | Very Low | Risk of bias=-1; Inconsistency=-1; Indirectness=-1; Imprecision=-1; Pub. bias: none | Map |
| Haaland 2008 (R04) | IL-6 | Reviewer-applied | Very Low | Risk of bias=-2; Inconsistency=-1; Indirectness=No; Imprecision=-2; Pub. bias: none | Core |
| Hejazi 2026 (R05) | IL-6 | Reviewer-applied | Very Low | Risk of bias=-1; Inconsistency=-2; Indirectness=No; Imprecision=-1; Pub. bias: none | Core |
| Khosravi 2019 (R06) | IL-6 | Reviewer-applied | Very Low | Risk of bias=-1; Inconsistency=-1; Indirectness=No; Imprecision=-1; Pub. bias: none | Core |
| Malandish & Gulati 2023 (R07) | IL-6 | Reviewer-applied | Moderate | Risk of bias=-1; Inconsistency=No/unclear; Indirectness=No; Imprecision=No serious concern; Pub. bias: none | Core |
| Mishra 2024 (R08) | IL-6 | Reviewer-applied | Very Low | Risk of bias=-2; Inconsistency=-2; Indirectness=-1; Imprecision=-1; Pub. bias: none | Map |
| Monteiro-Junior 2018 (R09) | IL-6 | Reviewer-applied | Very Low | Risk of bias=-1; Inconsistency=-2; Indirectness=No; Imprecision=No serious concern; Pub. bias: none | Core |
| Najafi 2022 (R10) | IL-6 | Reviewer-applied | Very Low | Risk of bias=-1; Inconsistency=-1; Indirectness=No; Imprecision=-2; Pub. bias: none | Core |
| Negaresh 2018 (R11) | IL-6 | Reviewer-applied | Very Low | Risk of bias=-1; Inconsistency=-1; Indirectness=No; Imprecision=-1; Pub. bias: none | Core |
| Palotino-Ferreira 2025 (R12) | IL-6 | Reviewer-applied | Very Low | Risk of bias=-1; Inconsistency=-1; Indirectness=-1; Imprecision=-2; Pub. bias: none | Map |
| Pedro 2017 (R13) | IL-6 | Reviewer-applied | Very Low | Risk of bias=-1; Inconsistency=-1; Indirectness=No; Imprecision=-2; Pub. bias: none | Core |
| Reis 2024 (R14) | IL-6 | Reviewer-applied | Very Low | Risk of bias=-2; Inconsistency=No/unclear; Indirectness=-2; Imprecision=-1; Pub. bias: none | Map |
| Sánchez-Romero 2026 (R15) | IL-6 | Reviewer-applied | Low | Risk of bias=No; Inconsistency=No/unclear; Indirectness=No; Imprecision=-2; Pub. bias: none | Core |
| Sánchez-Romero 2026 (R15) | IL-6 | SR-reported | Very Low | Risk of bias=No/low concern; Inconsistency=Low-Moderate; Indirectness=No; Imprecision=Serious; Publication bias=None | Core |
| Stigger 2019 (R16) | IL-6 | Reviewer-applied | Low | Risk of bias=-1; Inconsistency=No/unclear; Indirectness=No; Imprecision=-1; Pub. bias: none | Core |
| Teodoro 2025 (R17) | IL-6 | Reviewer-applied | Very Low | Risk of bias=-1; Inconsistency=-1; Indirectness=No; Imprecision=-2; Pub. bias: none | Core |
| Ubaghs 2026 (R18) | IL-6 | Reviewer-applied | Low | Risk of bias=-1; Inconsistency=No/unclear; Indirectness=No; Imprecision=-1; Pub. bias: none | Core |
| Wang 2024 (R19) | IL-6 | Reviewer-applied | Very Low | Risk of bias=-1; Inconsistency=-2; Indirectness=-1; Imprecision=-1; Pub. bias: none | Map |
| Wong & Holahan 2019 (R20) | IL-6 | Reviewer-applied | Very Low | Risk of bias=-2; Inconsistency=-1; Indirectness=No; Imprecision=-1; Pub. bias: none | Core |
| Zanetti 2021 (R21) | IL-6 | Reviewer-applied | Very Low | Risk of bias=-1; Inconsistency=-2; Indirectness=No; Imprecision=-2; Pub. bias: none | Core |
| Zanetti 2021 (R21) | IL-6 | SR-reported | Moderate | Risk of bias=Serious; Inconsistency=Not serious; Indirectness=Not serious; Imprecision=Not serious; Publication bias=Non | Core |
| Baker 2022 (R01) | TNF-alpha | Reviewer-applied | Very Low | Risk of bias=-1; Inconsistency=No/unclear; Indirectness=-1 (mixed adult/paediatric evidence); Imprecision=-1; Pub. bias: none | Map |
| Byrne 2023 (R02) | TNF-alpha | Reviewer-applied | Very Low | Risk of bias=-1; Inconsistency=-1; Indirectness=-1; Imprecision=-1; Pub. bias: none | Map |
| Hejazi 2026 (R05) | TNF-alpha | Reviewer-applied | Very Low | Risk of bias=-1; Inconsistency=-1; Indirectness=No; Imprecision=-2; Pub. bias: none | Core |
| Khosravi 2019 (R06) | TNF-alpha | Reviewer-applied | Low | Risk of bias=-1; Inconsistency=-1; Indirectness=No; Imprecision=No serious concern; Pub. bias: none | Core |
| Malandish & Gulati 2023 (R07) | TNF-alpha | Reviewer-applied | Very Low | Risk of bias=-1; Inconsistency=-1; Indirectness=No; Imprecision=-1; Pub. bias: none | Core |
| Mishra 2024 (R08) | TNF-alpha | Reviewer-applied | Very Low | Risk of bias=-2; Inconsistency=-1; Indirectness=-1; Imprecision=-1; Pub. bias: none | Map |
| Monteiro-Junior 2018 (R09) | TNF-alpha | Reviewer-applied | Very Low | Risk of bias=-1; Inconsistency=-2; Indirectness=No; Imprecision=-1; Pub. bias: none | Core |
| Najafi 2022 (R10) | TNF-alpha | Reviewer-applied | Very Low | Risk of bias=-1; Inconsistency=-1; Indirectness=No; Imprecision=-2; Pub. bias: none | Core |
| Negaresh 2018 (R11) | TNF-alpha | Reviewer-applied | Very Low | Risk of bias=-1; Inconsistency=-1; Indirectness=No; Imprecision=-1; Pub. bias: none | Core |
| Palotino-Ferreira 2025 (R12) | TNF-alpha | Reviewer-applied | Very Low | Risk of bias=-1; Inconsistency=-1; Indirectness=-1; Imprecision=-2; Pub. bias: none | Map |
| Pedro 2017 (R13) | TNF-alpha | Reviewer-applied | Very Low | Risk of bias=-1; Inconsistency=-1; Indirectness=No; Imprecision=-2; Pub. bias: none | Core |
| Reis 2024 (R14) | TNF-alpha | Reviewer-applied | Very Low | Risk of bias=-2; Inconsistency=No/unclear; Indirectness=-2; Imprecision=-1; Pub. bias: none | Map |
| Sánchez-Romero 2026 (R15) | TNF-alpha | Reviewer-applied | Low | Risk of bias=No; Inconsistency=No/unclear; Indirectness=No; Imprecision=-2; Pub. bias: none | Core |
| Sánchez-Romero 2026 (R15) | TNF-alpha | SR-reported | Very Low | Risk of bias=No/low concern; Inconsistency=Moderate; Indirectness=No; Imprecision=Serious; Publication bias=None | Core |
| Stigger 2019 (R16) | TNF-alpha | Reviewer-applied | Very Low | Risk of bias=-1; Inconsistency=-2; Indirectness=No; Imprecision=-1; Pub. bias: none | Core |
| Teodoro 2025 (R17) | TNF-alpha | Reviewer-applied | Very Low | Risk of bias=-1; Inconsistency=-1; Indirectness=No; Imprecision=-2; Pub. bias: none | Core |
| Ubaghs 2026 (R18) | TNF-alpha | Reviewer-applied | Very Low | Risk of bias=-1; Inconsistency=-1; Indirectness=No; Imprecision=-1; Pub. bias: none | Core |
| Wang 2024 (R19) | TNF-alpha | Reviewer-applied | Very Low | Risk of bias=-1; Inconsistency=-2; Indirectness=-1; Imprecision=-1; Pub. bias: none | Map |
| Wong & Holahan 2019 (R20) | TNF-alpha | Reviewer-applied | Very Low | Risk of bias=-2; Inconsistency=-1; Indirectness=No; Imprecision=-1; Pub. bias: none | Core |
| Zanetti 2021 (R21) | TNF-alpha | Reviewer-applied | Very Low | Risk of bias=-1; Inconsistency=-1; Indirectness=No; Imprecision=-2; Pub. bias: none | Core |
| Zanetti 2021 (R21) | TNF-alpha | SR-reported | Moderate | Risk of bias=Serious; Inconsistency=Not serious; Indirectness=Not serious; Imprecision=Not serious; Publication bias=Non | Core |
| Baker 2022 (R01) | CRP | Reviewer-applied | Very Low | Risk of bias=-1; Inconsistency=No/unclear; Indirectness=-1 (mixed adult/paediatric evidence); Imprecision=-1; Pub. bias: none | Map |
| Byrne 2023 (R02) | CRP | Reviewer-applied | Very Low | Risk of bias=-1; Inconsistency=-1; Indirectness=-1; Imprecision=-1; Pub. bias: none | Map |
| Haaland 2008 (R04) | CRP | Reviewer-applied | Very Low | Risk of bias=-2; Inconsistency=-1; Indirectness=No; Imprecision=-2; Pub. bias: none | Core |
| Khosravi 2019 (R06) | CRP | Reviewer-applied | Low | Risk of bias=-1; Inconsistency=-1; Indirectness=No; Imprecision=No serious concern; Pub. bias: none | Core |
| Mishra 2024 (R08) | CRP | Reviewer-applied | Very Low | Risk of bias=-2; Inconsistency=-2; Indirectness=-1; Imprecision=-1; Pub. bias: none | Map |
| Monteiro-Junior 2018 (R09) | CRP | Reviewer-applied | Very Low | Risk of bias=-1; Inconsistency=-2; Indirectness=No; Imprecision=No serious concern; Pub. bias: none | Core |
| Najafi 2022 (R10) | CRP | Reviewer-applied | Very Low | Risk of bias=-1; Inconsistency=-1; Indirectness=No; Imprecision=-2; Pub. bias: none | Core |
| Reis 2024 (R14) | CRP | Reviewer-applied | Very Low | Risk of bias=-2; Inconsistency=No/unclear; Indirectness=-2; Imprecision=-1; Pub. bias: none | Map |
| Stigger 2019 (R16) | CRP | Reviewer-applied | Very Low | Risk of bias=-1; Inconsistency=-1; Indirectness=No; Imprecision=-2; Pub. bias: none | Core |
| Ubaghs 2026 (R18) | CRP | Reviewer-applied | Very Low | Risk of bias=-1; Inconsistency=-2; Indirectness=No; Imprecision=-1; Pub. bias: none | Core |
| Wang 2024 (R19) | CRP | Reviewer-applied | Very Low | Risk of bias=-1; Inconsistency=-2; Indirectness=-1; Imprecision=-2; Pub. bias: none | Map |
| Malandish & Gulati 2023 (R07) | hs-CRP | Reviewer-applied | Low | Risk of bias=-1; Inconsistency=-1; Indirectness=No; Imprecision=No serious concern; Pub. bias: none | Core |
| Pedro 2017 (R13) | hs-CRP | Reviewer-applied | Very Low | Risk of bias=-1; Inconsistency=-1; Indirectness=No; Imprecision=-2; Pub. bias: none | Core |
| Khosravi 2019 (R06) | IL-8 | Reviewer-applied | Very Low | Risk of bias=-1; Inconsistency=-1; Indirectness=No; Imprecision=-1; Pub. bias: none | Core |
| Malandish & Gulati 2023 (R07) | IL-8 | Reviewer-applied | Very Low | Risk of bias=-1; Inconsistency=-1; Indirectness=No; Imprecision=-1; Pub. bias: none | Core |
| Sánchez-Romero 2026 (R15) | IL-8 | Reviewer-applied | Low | Risk of bias=No; Inconsistency=No/unclear; Indirectness=No; Imprecision=-2; Pub. bias: none | Core |
| Sánchez-Romero 2026 (R15) | IL-8 | SR-reported | Very Low | Risk of bias=No/low concern; Inconsistency=Serious; Indirectness=No; Imprecision=Very serious; Publication bias=None | Core |
| Malandish & Gulati 2023 (R07) | IL-1beta | Reviewer-applied | Very Low | Risk of bias=-1; Inconsistency=-1; Indirectness=No; Imprecision=-1; Pub. bias: none | Core |
| Stigger 2019 (R16) | IL-1beta | Reviewer-applied | Very Low | Risk of bias=-1; Inconsistency=-1; Indirectness=No; Imprecision=-2; Pub. bias: none | Core |
| Khosravi 2019 (R06) | CCL2/MCP-1 | Reviewer-applied | Very Low | Risk of bias=-1; Inconsistency=No/unclear; Indirectness=No; Imprecision=-2; Pub. bias: none | Core |
| Sánchez-Romero 2026 (R15) | CCL2/MCP-1 | Reviewer-applied | Low | Risk of bias=No; Inconsistency=No/unclear; Indirectness=No; Imprecision=-2; Pub. bias: none | Core |
| Sánchez-Romero 2026 (R15) | CCL2/MCP-1 | SR-reported | Very Low | Risk of bias=No/low concern; Inconsistency=Serious; Indirectness=No; Imprecision=Very serious; Publication bias=None | Core |
| Chen 2021 (R03) | p16INK4a | Reviewer-applied | Very Low | Risk of bias=-1; Inconsistency=-1; Indirectness=-1; Imprecision=-1; Pub. bias: none | Map |
| Chen 2021 (R03) | p21Cip1 | Reviewer-applied | Very Low | Risk of bias=-1; Inconsistency=-1; Indirectness=-1; Imprecision=-2; Pub. bias: none | Map |
| Haaland 2008 (R04) | IL-18 | Reviewer-applied | Very Low | Risk of bias=-2; Inconsistency=-1; Indirectness=No; Imprecision=-2; Pub. bias: none | Core |
| Haaland 2008 (R04) | TNF-alpha / soluble TNF receptors | Reviewer-applied | Very Low | Risk of bias=-2; Inconsistency=-1; Indirectness=No; Imprecision=-2; Pub. bias: none | Core |
| Pedro 2017 (R13) | IL-18 | Reviewer-applied | Very Low | Risk of bias=-1; Inconsistency=-1; Indirectness=No; Imprecision=-2; Pub. bias: none | Core |

GRADE, Grading of Recommendations Assessment, Development and Evaluation. Source: SR-reported (certainty as graded by the source review) or Reviewer-applied (certainty graded for this umbrella review from the information available in the source review). Tier: Core = core-eligible; Map = evidence-map-only.

Downgrade considerations summarize the GRADE domains (risk of bias, inconsistency, indirectness, imprecision, publication bias) that reduced certainty. The construct-level summary (most conservative grade and range) is given in Main Table 4. Counts of frequent downgrade domains in Main Table 4 are given as the number of review–construct assessment entries in which the domain was downgraded, out of the total number of assessment entries for that construct (IL-6, 22; TNF-alpha, 21; CRP, 11; IL-8, 4; CCL2/MCP-1, 3; hs-CRP, IL-1beta and IL-18, 2 each; p16INK4a, p21Cip1 and TNF-alpha/soluble TNF receptors, 1 each). A review contributes more than one entry when both a source-reported and a reviewer-applied certainty rating were available, so these denominators can exceed the number of contributing reviews. A domain was counted as downgraded when the entry recorded a downgrade (reviewer-applied ratings) or a moderate, serious or very serious concern (source-reported ratings). Each review contributes at most one reviewer-applied row per construct in this table. Where a review reported more than one within-review sub-estimate for a construct (for example, an acute-versus-chronic timepoint split, or a primary analysis alongside a sensitivity analysis), the row shown reflects the most conservative certainty grade and the most conservative rating in each GRADE domain across those sub-estimates, consistent with the construct-level presentation described in Methods. Disaggregated sub-estimates remain available in Supplementary Table S2 and, for exercise-modality subgroups, in Supplementary Table S7 and Figure S1.

**Table S5. Post hoc restricted descriptive sensitivity analysis excluding core-eligible reviews rated critically low on AMSTAR-2.**

| **Review** | **AMSTAR-2 confidence** | **Condition / population** | **Outcome** | **Metric** | **Effect [95% interval]** | **I²** | **Retained in restricted synthesis** |
| --- | --- | --- | --- | --- | --- | --- | --- |
| Hejazi 2026 (R05) | Low | Multiple sclerosis | IL-6 | SMD | +0.09 [−0.17, +0.36] | 92% | Yes |
| Hejazi 2026 (R05) | Low | Multiple sclerosis | TNF-alpha | SMD | −0.31 [−0.56, −0.06] | 72% | Yes |
| Malandish & Gulati 2023 (R07) | Low | Heart failure with overweight/obesity | IL-6 | SMD | −0.20 [−0.33, −0.08] | 9% | Yes |
| Malandish & Gulati 2023 (R07) | Low | Heart failure with overweight/obesity | TNF-alpha | SMD | −0.06 [−0.31, +0.19] | 75% | Yes |
| Malandish & Gulati 2023 (R07) | Low | Heart failure with overweight/obesity | hs-CRP | SMD | −0.38 [−0.56, −0.20] | 74% | Yes |
| Sánchez-Romero 2026 (R15) | Moderate | SASP older adults | IL-6 | MD | −0.04 [−0.97, +0.91] | NR | Yes |
| Sánchez-Romero 2026 (R15) | Moderate | SASP older adults | TNF-alpha | MD | −0.03 [−1.44, +1.38] | NR | Yes |
| Stigger 2019 (R16) | Low | Dementia/MCI | IL-6 | MD | −0.71 [−0.88, −0.54] | 0% | Yes |
| Stigger 2019 (R16) | Low | Dementia/MCI | TNF-alpha | MD | −1.00 [−1.90, −0.10] | 90% | Yes |
| Teodoro 2025 (R17) | Low | Older adults (TH1/TH2) | IL-6; TNF-alpha | Narrative | No pooled estimate reported | NA | Yes (narrative only) |
| Ubaghs 2026 (R18) | Low | Breast cancer | IL-6 | SMD | −0.01 [−0.24, +0.21] | 27% | Yes |
| Ubaghs 2026 (R18) | Low | Breast cancer | TNF-alpha | SMD | −0.05 [−0.57, +0.47] | 78% | Yes |
| Ubaghs 2026 (R18) | Low | Breast cancer | CRP | SMD | −0.63 [−1.51, +0.25] | 89% | Yes |
| Zanetti 2021 (R21) | Low | HIV | IL-6 | MD | −1.18 [−3.71, +1.35] | 93% | Yes |
| Zanetti 2021 (R21) | Low | HIV | TNF-alpha | MD | −4.76 [−10.81, +1.29] | 68% | Yes |
| Haaland 2008 (R04) | Critically Low | Healthy older adults | IL-6; TNF-alpha and soluble TNF receptors; CRP; IL-18 | Narrative | No pooled estimate reported | NA | No |
| Khosravi 2019 (R06) | Critically Low | Cancer | IL-6 | SMD | −0.16 [−0.38, +0.06] | 38% | No |
| Khosravi 2019 (R06) | Critically Low | Cancer | TNF-alpha | SMD | −0.30 [−0.53, −0.06] | 52% | No |
| Khosravi 2019 (R06) | Critically Low | Cancer | CRP | SMD | −0.47 [−0.87, −0.06] | 67% | No |
| Monteiro-Junior 2018 (R09) | Critically Low | General older adults | IL-6 | SMD | −0.81 [−1.39, −0.23] | 90% | No |
| Monteiro-Junior 2018 (R09) | Critically Low | General older adults | TNF-alpha | SMD | −1.42 [−2.96, +0.13] | 94% | No |
| Monteiro-Junior 2018 (R09) | Critically Low | General older adults | CRP | SMD | −0.65 [−1.11, −0.18] | 87% | No |
| Najafi 2022 (R10) | Critically Low | Multiple sclerosis | IL-6; TNF-alpha; CRP | Narrative | No pooled estimate reported | NA | No |
| Negaresh 2018 (R11) | Critically Low | Multiple sclerosis | IL-6; TNF-alpha | Narrative | No pooled estimate reported | NA | No |
| Pedro 2017 (R13) | Critically Low | HIV | IL-6; TNF-alpha; hs-CRP; IL-18 | Narrative | No pooled estimate reported | NA | No |
| Wong & Holahan 2019 (R20) | Critically Low | Multiple sclerosis | IL-6; TNF-alpha | Narrative | No pooled estimate reported | NA | No |

CI, confidence interval; CRP, C-reactive protein; hs-CRP, high-sensitivity CRP; IBD, inflammatory bowel disease; IL, interleukin; MCI, mild cognitive impairment; MD, mean difference; SASP, senescence-associated secretory phenotype; SMD, standardized mean difference; TNF-alpha, tumour necrosis factor alpha; I², heterogeneity statistic; 95% interval, confidence or credible interval as reported by the source review; NR, not reported by the source review; NA, not applicable or not estimable. A negative estimate favours exercise.

Only the 14 core-eligible reviews are listed. Seven critically low reviews were excluded from the restricted description and seven reviews were retained (one moderate and six low confidence), including one review contributing narrative evidence only. Estimates are reproduced from source reviews and are not re-pooled. AMSTAR-2 is used here as a review-level restriction and not as a substitute for primary-study risk-of-bias assessment.

Restricted-set SMD ranges: IL-6 −0.20 to +0.09 (one of three intervals excluding the null); TNF-alpha −0.31 to −0.05 (one of three); CRP −0.63 (interval crossing the null); hs-CRP −0.38 (interval excluding the null). Full-set core-eligible SMD ranges for comparison: IL-6 −0.81 to +0.09 (two of five); TNF-alpha −1.42 to −0.05 (two of five); CRP −0.65 to −0.47 (two of three). This analysis is post hoc, descriptive and hypothesis-generating; it does not provide a pooled effect or upgrade certainty.

**Table S6. Primary-study overlap between reviews (corrected covered area).**

| **Cluster** | **Reviews (n)** | **Deduplicated primary studies (n)** | **CCA** | **Overlap level** | **Notable pairwise overlaps (shared studies)** |
| --- | --- | --- | --- | --- | --- |
| Overall | 20 | 249 | 0.0091 | slight | R04-R09: 1; R04-R17: 1; R05-R10: 13; R05-R11: 8; R05-R20: 8; R06-R08: 1; R06-R18: 3; R07-R08: 1; R09-R17: 1; R09-R19: 1; R10-R11: 12; R10-R20: 9; R11-R20: 8 |
| Multiple sclerosis | 4 | 28 | 0.4048 | very high | R05-R10: 13; R05-R11: 8; R05-R20: 8; R10-R11: 12; R10-R20: 9; R11-R20: 8 |
| Cancer | 2 | 42 | 0.0714 | moderate | R06-R18: 3 |
| HIV | 2 | 11 | 0.0000 | slight | No non-zero pairwise overlap. |
| Older adults/general aging | 5 | 47 | 0.0160 | slight | R04-R09: 1; R04-R17: 1; R09-R17: 1 |
| Whole-body vibration sensitivity | 2 | 23 | 0.0435 | slight | R09-R19: 1 |
| Yoga/mind-body sensitivity | 2 | 71 | 0.0141 | slight | R07-R08: 1 |

CCA, corrected covered area, an overlap metric ranging from 0 (no overlap) upward; interpretive bands: slight (0–5%), moderate (>5–10%), high (>10–15%) and very high (>15%).

Overlap was computed from the study-by-review citation grid. Pairwise cells give the number of primary studies shared by a pair of reviews; the full pairwise matrix is shown as a heatmap in the figures and is not repeated here. Overall overlap is slight, but overlap within the multiple-sclerosis cluster is very high, which is why review-level estimates in that cluster are not pooled.

**Table S7. Source-reported exercise-modality subgroup analyses.**

| **Review** | **Outcome** | **Exercise-modality subgroup** | **Metric** | **Effect [95% CI]** | **I²** | **k** |
| --- | --- | --- | --- | --- | --- | --- |
| Hejazi 2026 (R05) | IL-6 | aerobic training (AT) | SMD | +0.30 [-0.04, +0.63] | 94% | 7 arms |
| Hejazi 2026 (R05) | IL-6 | concurrent aerobic + resistance training (AT + RT) | SMD | -0.39 [-0.81, +0.04] | 51% | 3 arms |
| Hejazi 2026 (R05) | TNF-alpha | aerobic training (AT) | SMD | -0.56 [-1.00, -0.12] | 76% | 4 arms |
| Hejazi 2026 (R05) | TNF-alpha | concurrent aerobic + resistance training (AT + RT) | SMD | +0.13 [-0.27, +0.53] | 60% | 3 arms |
| Hejazi 2026 (R05) | TNF-alpha | resistance training (RT) | SMD | -0.25 [-0.97, +0.47] | NA | 1 arm |
| Hejazi 2026 (R05) | TNF-alpha | high-intensity interval training (HIIT) | SMD | -1.04 [-1.71, -0.37] | NA | 1 arm |

CI, confidence interval; k, number of contributing arms/studies; SMD, standardized mean difference; TNF-alpha, tumour necrosis factor alpha; I², heterogeneity statistic; NA, not applicable or not estimable.

This table reproduces exercise-modality subgroups from a single systematic review (Hejazi 2026; multiple sclerosis). These estimates are hypothesis-generating and are not an umbrella-level meta-regression. Single-arm subgroups have no heterogeneity estimate (I²), although the source review reported an effect estimate and 95% confidence interval. A negative estimate favours exercise.

**Supplementary Figure**

**Figure S1. Exercise-modality subgroup analyses reported within a single review.**

Exercise-modality subgroups for IL-6 and TNF-alpha reported within Hejazi 2026 (multiple sclerosis). Squares are source-reported subgroup standardized mean differences and whiskers are 95% confidence intervals. Single-arm subgroups have no heterogeneity estimate (I²), although the source review reported an effect estimate and confidence interval. These subgroup findings are hypothesis-generating and are not an umbrella-level meta-regression. A negative value favours exercise. CI, confidence interval; SMD, standardized mean difference; TNF-alpha, tumour necrosis factor alpha.

**PRISMA 2020 and PRIOR checklist: item-to-section mapping**

The tables below map each PRISMA 2020 and PRIOR checklist item to the final submission files. Locations refer to the line-numbered revised Main Manuscript and the final Supplementary Table and Figure numbering.

**PRISMA 2020 checklist**

| **Section / Topic** | **Item** | **Checklist item (abbreviated)** | **Location (section; page/line)** |
| --- | --- | --- | --- |
| Title | 1 | Identify the report as a systematic review (umbrella review). | Main Manuscript, Title, p. 1, lines 1–3. |
| Abstract | 2 | Structured summary. | Main Manuscript, Abstract, pp. 1–2, lines 11–41. |
| Introduction | 3 | Rationale for the review. | Main Manuscript, Introduction, pp. 2–3, lines 45–91. |
| Introduction | 4 | Objectives / review question(s). | Main Manuscript, Introduction—objective, p. 3, lines 87–91. |
| Methods | 5 | Eligibility criteria. | Main Manuscript, Methods—Eligibility criteria, pp. 3–4, lines 111–143. |
| Methods | 6 | Information sources. | Main Manuscript, Methods—Information sources and search, p. 4, lines 144–151. |
| Methods | 7 | Search strategy (full strategies). | Main Manuscript, Methods—Information sources and search, p. 4, lines 144–151; Supplementary Table S1, pp. 1–2. |
| Methods | 8 | Selection process. | Main Manuscript, Methods—Study selection and data extraction, p. 4, lines 152–163; Results—Review selection, p. 6, lines 219–228. |
| Methods | 9 | Data collection process. | Main Manuscript, Methods—Study selection and data extraction, p. 4, lines 152–163. |
| Methods | 10a | Data items – outcomes. | Main Manuscript, Methods—Eligibility criteria, p. 4, lines 129–132; Supplementary Table S2, pp. 3–5. |
| Methods | 10b | Data items – other variables. | Main Manuscript, Methods—Study selection and data extraction, p. 4, lines 155–163; Supplementary Table S2, pp. 3–5. |
| Methods | 11 | Study (review) risk of bias assessment. | Main Manuscript, Methods—Methodological quality of included reviews, pp. 4–5, lines 164–172. |
| Methods | 12 | Effect measures. | Main Manuscript, Methods—Data synthesis, pp. 5–6, lines 196–218; Table 2, pp. 18–19. |
| Methods | 13a | Synthesis – eligibility for each synthesis. | Main Manuscript, Methods—Data synthesis, pp. 5–6, lines 196–218. |
| Methods | 13b | Synthesis – data preparation. | Main Manuscript, Methods—Study selection and data extraction and Data synthesis, pp. 4–6, lines 152–163 and 196–218. |
| Methods | 13c | Synthesis – tabulation / visual display. | Main Manuscript, Results and Tables/Figures; Supplementary Tables S2–S7 and Figure S1. |
| Methods | 13d | Synthesis – methods of synthesis. | Main Manuscript, Methods—Data synthesis, pp. 5–6, lines 196–218. |
| Methods | 13e | Synthesis – exploration of heterogeneity. | Main Manuscript, Methods—Data synthesis, pp. 5–6, lines 196–218; Results—Effects, p. 7, lines 255–278; Discussion, p. 9, lines 358–367. |
| Methods | 13f | Synthesis – sensitivity analyses. | Main Manuscript, Results—Post hoc restricted descriptive sensitivity analysis, p. 7, lines 288–299; Subgroup and sensitivity analyses, p. 8, lines 319–329; Supplementary Tables S2, S5 and S7. |
| Methods | 14 | Reporting bias assessment. | Main Manuscript, Methods—Certainty of evidence and Data synthesis, pp. 5–6, lines 180–218; Results—Methodological quality, p. 7, lines 279–287. |
| Methods | 15 | Certainty assessment (GRADE). | Main Manuscript, Methods—Certainty of evidence, p. 5, lines 180–195; Supplementary Table S4, pp. 7–8. |
| Results | 16a | Study selection – flow. | Main Manuscript, Results—Review selection, p. 6, lines 219–228; Figure 1 and its legend, p. 25, lines 599–603. |
| Results | 16b | Study selection – excluded with reasons. | Main Manuscript, Results—Review selection, p. 6, lines 219–228; Figure 1 and its legend, p. 25, lines 599–603. |
| Results | 17 | Study characteristics. | Main Manuscript, Results—Characteristics, p. 6, lines 229–243; Table 1, pp. 15–17; Supplementary Table S3, p. 6. |
| Results | 18 | Risk of bias in studies (reviews). | Main Manuscript, Results—Methodological quality, p. 7, lines 279–287; Figure 4 and its legend, p. 25, lines 624–630; Table 3, pp. 20–21. |
| Results | 19 | Results of individual studies (reviews). | Main Manuscript, Results—Effects on core biomarkers, p. 7, lines 255–278; Table 2, pp. 18–19; Figure 3 and its legend, p. 25, lines 613–622. |
| Results | 20a | Results of syntheses – characteristics/risk of bias. | Main Manuscript, Results—Effects, Methodological quality, Restricted analysis and Certainty, pp. 7–8, lines 255–307; Tables 2–4; Supplementary Tables S4–S5. |
| Results | 20b | Results of syntheses – statistical results. | Main Manuscript, Results—Effects, p. 7, lines 255–278; Table 2, pp. 18–19. |
| Results | 20c | Results of syntheses – heterogeneity. | Main Manuscript, Results—Effects, p. 7, lines 255–278; Table 2, pp. 18–19. |
| Results | 20d | Results of syntheses – sensitivity. | Main Manuscript, Results—Post hoc restricted descriptive sensitivity analysis, p. 7, lines 288–299; Subgroup and sensitivity analyses, p. 8, lines 319–329; Supplementary Tables S2, S5 and S7. |
| Results | 21 | Reporting biases. | Main Manuscript, Results—Methodological quality, p. 7, lines 279–287; Table 3, pp. 20–21. |
| Results | 22 | Certainty of evidence. | Main Manuscript, Results—Certainty of the evidence, p. 8, lines 300–307; Table 4, pp. 22–24; Supplementary Table S4, pp. 7–8. |
| Discussion | 23a | Interpretation. | Main Manuscript, Discussion, pp. 8–9, lines 330–377. |
| Discussion | 23b | Limitations of the evidence. | Main Manuscript, Strengths and limitations, pp. 9–10, lines 378–393. |
| Discussion | 23c | Limitations of the review processes. | Main Manuscript, Strengths and limitations, pp. 9–10, lines 378–393. |
| Discussion | 23d | Implications. | Main Manuscript, Implications for research and practice, p. 10, lines 394–406. |
| Other | 24a | Registration and protocol. | Main Manuscript, Methods—Protocol, registration and reporting, p. 3, lines 94–110. |
| Other | 24b | Protocol access. | Main Manuscript, Methods—Protocol, registration and reporting, p. 3, lines 94–110 (PROSPERO CRD420261410061). |
| Other | 24c | Amendments to protocol. | Main Manuscript, Methods—Protocol, registration and reporting, p. 3, lines 94–110; protocol amendments are described explicitly. |
| Other | 25 | Support / funding. | Main Manuscript, Funding, p. 11, lines 431–433. |
| Other | 26 | Competing interests. | Main Manuscript, Conflict of interest, p. 11, lines 434–436. |
| Other | 27 | Availability of data, code and materials. | Main Manuscript, Data availability statement, p. 10, lines 419–421; Supplementary Tables S1–S7 and Figure S1. |

**PRIOR checklist (overviews of reviews)**

| **Section / Topic** | **Item** | **Checklist item (abbreviated)** | **Location (section; page/line)** |
| --- | --- | --- | --- |
| Title | 1 | Identify the report as an overview/umbrella review of systematic reviews. | Main Manuscript, Title, p. 1, lines 1–3. |
| Abstract | 2 | Structured summary appropriate to an overview. | Main Manuscript, Abstract, pp. 1–2, lines 11–41. |
| Introduction | 3 | Rationale for conducting an overview of reviews. | Main Manuscript, Introduction, pp. 2–3, lines 45–91. |
| Introduction | 4 | Objectives, with reference to the overview question. | Main Manuscript, Introduction—objective, p. 3, lines 87–91. |
| Methods | 5 | Eligibility criteria for including systematic reviews. | Main Manuscript, Methods—Eligibility criteria, pp. 3–4, lines 111–143. |
| Methods | 6 | Information sources. | Main Manuscript, Methods—Information sources and search, p. 4, lines 144–151. |
| Methods | 7 | Search strategy. | Main Manuscript, Methods—Information sources and search, p. 4, lines 144–151; Supplementary Table S1, pp. 1–2. |
| Methods | 8 | Selection process for systematic reviews. | Main Manuscript, Methods—Study selection and data extraction, p. 4, lines 152–163; Results—Review selection, p. 6, lines 219–228. |
| Methods | 9 | Data collection process from reviews. | Main Manuscript, Methods—Study selection and data extraction, p. 4, lines 152–163. |
| Methods | 10 | Data items collected from the included reviews. | Main Manuscript, Methods—Study selection and data extraction, p. 4, lines 155–163; Supplementary Table S2, pp. 3–5. |
| Methods | 11 | Assessment of methodological quality of included reviews. | Main Manuscript, Methods—Methodological quality of included reviews, pp. 4–5, lines 164–172. |
| Methods | 12 | Assessment and management of primary-study overlap between reviews. | Main Manuscript, Methods—Primary-study overlap, p. 5, lines 173–179; Results—overlap, p. 6, lines 244–253; Supplementary Table S6, p. 10. |
| Methods | 13 | Effect measures and handling of estimates across reviews. | Main Manuscript, Methods—Data synthesis, pp. 5–6, lines 196–218; Table 2, pp. 18–19. |
| Methods | 14 | Synthesis methods, including decision not to re-pool overlapping reviews. | Main Manuscript, Methods—Data synthesis, pp. 5–6, lines 196–218. |
| Methods | 15 | Assessment of certainty of the evidence. | Main Manuscript, Methods—Certainty of evidence, p. 5, lines 180–195; Supplementary Table S4, pp. 7–8. |
| Methods | 16 | Handling of discordant or overlapping review findings. | Main Manuscript, Methods—Primary-study overlap and Data synthesis, pp. 5–6, lines 173–218; Results—overlap, p. 6, lines 244–253. |
| Results | 17 | Selection of reviews (flow). | Main Manuscript, Results—Review selection, p. 6, lines 219–228; Figure 1 and its legend, p. 25, lines 599–603. |
| Results | 18 | Characteristics of the included reviews. | Main Manuscript, Results—Characteristics, p. 6, lines 229–243; Table 1, pp. 15–17; Supplementary Table S3, p. 6. |
| Results | 19 | Methodological quality of the included reviews. | Main Manuscript, Results—Methodological quality, p. 7, lines 279–287; Figure 4 and its legend, p. 25, lines 624–630; Table 3, pp. 20–21. |
| Results | 20 | Primary-study overlap between reviews. | Main Manuscript, Results—Primary-study overlap, p. 6, lines 244–253; Figure 2 and its legend, p. 25, lines 605–611; Supplementary Table S6, p. 10. |
| Results | 21 | Results of the syntheses across reviews. | Main Manuscript, Results—Effects, Restricted analysis, Evidence map and Subgroup/sensitivity analyses, pp. 7–8, lines 255–329; Tables 2 and 4; Figures 3 and 5; Supplementary Tables S2, S5 and S7. |
| Results | 22 | Certainty of the evidence. | Main Manuscript, Results—Certainty of the evidence, p. 8, lines 300–307; Table 4, pp. 22–24; Supplementary Table S4, pp. 7–8. |
| Discussion | 23 | Summary, limitations and implications. | Main Manuscript, Discussion, Strengths and limitations, and Implications, pp. 8–10, lines 330–406. |
| Other | 24 | Registration and protocol. | Main Manuscript, Methods—Protocol, registration and reporting, p. 3, lines 94–110 (PROSPERO CRD420261410061). |
| Other | 25 | Support / funding. | Main Manuscript, Funding, p. 11, lines 431–433. |
| Other | 26 | Competing interests. | Main Manuscript, Conflict of interest, p. 11, lines 434–436. |
| Other | 27 | Availability of data, code and materials. | Main Manuscript, Data availability statement, p. 10, lines 419–421; Supplementary Tables S1–S7 and Figure S1. |
